# Analytical estimation of maximum fraction of infected individuals with one-shot non-pharmaceutical intervention in a hybrid epidemic model

**DOI:** 10.1186/s12879-022-07403-5

**Published:** 2022-06-01

**Authors:** Naoya Fujiwara, Tomokatsu Onaga, Takayuki Wada, Shouhei Takeuchi, Junji Seto, Tomoki Nakaya, Kazuyuki Aihara

**Affiliations:** 1 Graduate School of Information Sciences, Tohoku University, 6-3-09 Aoba, Aramaki-aza Aoba-ku, Sendai, 980-8579 Miyagi Japan; 2 PRESTO, Japan Science and Technology Agency (JST), 4-1-8 Honcho, Kawaguchi, 332-0012 Saitama Japan; 3 Institute of Industrial Science, The University of Tokyo, 4-6-1 Komaba, Meguro-ku, 153-8505 Tokyo Japan; 4 Center for Spatial Information Science, The University of Tokyo, 5-1-5 Kashiwanoha, Kashiwa, 277-8508 Chiba Japan; 5 Frontier Research Institute for Interdisciplinary Sciences, Tohoku University, Aramaki aza Aoba 6-3, Aoba-ku, Sendai, 980-8578 Miyagi Japan; 6 Department of Microbiology, Graduate School of Human Life and Ecology, Osaka Metropolitan University, 3-3-138, Sugimoto, Sumiyoshi-ku, Osaka, 558-8585 Osaka Japan; 7 Faculty of Nursing and Nutrition, University of Nagasaki, 1-1-1 Manabino, Nagayo-cho, Nishi-Sonogi-gun, Nagasaki, 851-2195 Japan; 8 Department of Microbiology, Yamagata Prefectural Institute of Public Health, 1-6-6 Toka-machi, Yamagata, 990-0031 Yamagata Japan; 9 Graduate School of Environmental Studies, Tohoku University, Aoba, 468-1, Aramaki, Aoba-ku, Sendai, 980-8572 Miyagi Japan; 10 International Research Center for Neurointelligence, The University of Tokyo, 7-3-1 Hongo, Bunkyo-ku, 113-0033 Tokyo Japan

**Keywords:** Infectious diseases, Pandemics, Non-pharmaceutical interventions, Hybrid dynamical systems

## Abstract

**Background:**

Facing a global epidemic of new infectious diseases such as COVID-19, non-pharmaceutical interventions (NPIs), which reduce transmission rates without medical actions, are being implemented around the world to mitigate spreads. One of the problems in assessing the effects of NPIs is that different NPIs have been implemented at different times based on the situation of each country; therefore, few assumptions can be shared about how the introduction of policies affects the patient population. Mathematical models can contribute to further understanding these phenomena by obtaining analytical solutions as well as numerical simulations.

**Methods and results:**

In this study, an NPI was introduced into the SIR model for a conceptual study of infectious diseases under the condition that the transmission rate was reduced to a fixed value only once within a finite time duration, and its effect was analyzed numerically and theoretically. It was analytically shown that the maximum fraction of infected individuals and the final size could be larger if the intervention starts too early. The analytical results also suggested that more individuals may be infected at the peak of the second wave with a stronger intervention.

**Conclusions:**

This study provides quantitative relationship between the strength of a one-shot intervention and the reduction in the number of patients with no approximation. This suggests the importance of the strength and time of NPIs, although detailed studies are necessary for the implementation of NPIs in complicated real-world environments as the model used in this study is based on various simplifications.

**Supplementary Information:**

The online version contains supplementary material available at 10.1186/s12879-022-07403-5.

## Introduction

Because of the global spread of COVID-19, our human society is facing a major public health crisis. The COVID-19 pandemic is caused by an emerging pathogen, SARS-CoV-2, for which there is no immunized population, causing an overshooting increase in the number of infected patients and depleting medical resources in many countries. Medical institutions are facing a difficult situation in which they must control second transmissions while treating critically ill patients, and as the number of patients increases, the medical system becomes swiftly tighter. When the number of patients exceeds the capacity, the quality of medical care deteriorates drastically, and the number of medical devices required for life support reaches its limit. This situation further increases the fatality rate of this infectious disease and causes serious damage to our society.

No effective treatment for COVID-19 has been established yet as of September, 2020, and only a public health approach can function as a control measure for the epidemic. To mitigate the spread of COVID-19, each country is implementing non-pharmaceutical interventions (NPIs) [1] to regulate social activities. NPIs comprise policies such as case isolation, voluntary home quarantine, closure of schools and universities, social distancing, stopping mass gatherings, and border closure. In major European countries, these NPIs were implemented, depending on the epidemic situation, during the first part of spring in 2020, with different timings and intensities [2, 3]. Although the first phase of the epidemic appeared to be suppressed by these mitigation measures, the re-epidemic became clearer in almost all countries because of deregulation after the first epidemic.

Because the control of epidemics by NPIs has caused a situation involving the imposition of strong restrictions on human socioeconomic activities, it would be desirable to study in advance the optimal timing, intensity, and duration of interventions that could bring about more promising results with minimal damage to society. Regarding COVID-19, for the purpose of ex-post verification, the influence of NPIs implemented in each country on the effective reproduction number was estimated. In European countries, the correlation between the decay of the effective reproduction number and the implementation of various NPIs has been verified [3]. The impact of travel limitations in China on the spread of infection has been discussed [4, 5]. However, a reliable estimation of the effects of NPIs is difficult, because the differences in NPI strategies employed by each country are strongly related to various background factors, such as the epidemic situations, social structure, legal systems, and culture [2].

Mathematical modelling is an important method for estimating the effect of NPIs. In particular, the global pandemic of COVID-19 revealed that a situation in which only NPIs are effective against an emerging infectious disease is possible in societies in the 2020s. Such a situation had been “neglected” as a practical research target, which enhances the importance of theoretical approaches. Recently, compartmental models such as the SIR model [6–9] have been extended to estimate the effects of NPIs on the number of patients [10–13]. In other settings, optimal policies where intervention intensity can change continuously over time have been discussed in the context of minimizing objective functions [14–17]. These solutions are very useful if we can estimate the effect of NPIs on the change in transmission parameters precisely.

The impacts of NPIs should be evaluated as a discontinuous change of the transmission rate in a model to represent the temporal discontinuity of intervention in the real world. The dynamics of the number of infected patients in continuous time in a system that includes discrete parameters, state spaces, and continuous-time dynamics can be modeled as a *hybrid dynamical system* [18–22]. This framework has been applied to mathematical models of infectious diseases [23–25]. In the simplest case, the *one-shot intervention* model, in which the intervention is implemented only once during the epidemic, can be used to discuss the theoretical dependence of the effects of NPIs on the timing and intensity [26]. As the COVID-19 epidemic continues, the accumulation of theoretical research on the effects brought about by NPIs has become even more significant. Recently, compartmental models with intervention have been studied both numerically [27, 28] and using some analytical methods [29, 30] in line with the COVID-19 epidemic.

In this study, we provide exact solutions of a simple SIR model with one-shot intervention, represented by a single discrete reduction in the transmission parameter during an epidemic. These solutions describe the dependence of the peak number of infected patients on the reproduction number under consideration of the implementation of NPIs and intervention timing. Theoretical and numerical analyses revealed non-trivial relations among the intensity of suppression of pandemics via NPIs, the number of infected individuals at the peaks, and the final size of infection cases.

The methods and results shown in this study provide basic theoretical understanding in the context of the evaluation of NPIs.

## Materials and methods

In this study, we focus on the dependence of the maximum fraction of infected individuals on the timing of the NPIs. Note that this analysis is motivated by the COVID-19 epidemic, but we consider a hypothetical epidemic, whose properties are not necessarily the same as that of COVID-19.

### Model

We here introduce the SIR model, where the time evolution of the fraction of susceptible (*s*(*t*)), infected (*i*(*t*)), and removed (*r*(*t*)) individuals is given by the following ordinary differential equation (ODE):

1 $$\begin{aligned} \frac{ds}{dt}= & {} -\beta s i, \end{aligned}$$

2 $$\begin{aligned} \frac{di}{dt}= & {} \beta s i - \gamma i, \end{aligned}$$

3 $$\begin{aligned} \frac{dr}{dt}= & {} \gamma i, \end{aligned}$$

taking into account the one-shot intervention and the second wave after the intervention. For simplicity, the total population is assumed to be unity, that is, $s(t)+i(t)+r(t)=1$ holds. The summation of the right-hand sides of Eqs. (1)–(3) vanishes, which guarantees conservation of the total population. Therefore, as seen below, only Eqs. (1) and (2) are numerically integrated to obtain the time evolutions of *s*(*t*), *i*(*t*), and *r*(*t*). In this model, the basic reproduction number and the effective reproduction number at time *t* are given by $R_0 = \beta /\gamma$ and $R_t = \beta s(t) / \gamma$, respectively.

Kermack and McKendrick [6] derived the equation for the maximum fraction of infected individuals and showed that the final size is obtained by solving a transcendental equation with a given $R_0$ in the SIR model without any intervention [7–9]. By applying the technique they employed in the derivation, we here provide the relationship between the fractions of susceptible and removed individuals at arbitrary times $t_0$ and $t_1$, that is, $s(t_0)$, $r(t_0)$, and $s(t_1)$, $r(t_1)$ as

4 $$\begin{aligned} s(t_1)= & {} s(t_0) \exp \bigg \{ - \frac{\beta }{\gamma } [r(t_1)-r(t_0)] \bigg \}. \end{aligned}$$

See Additional file 1: Section S1 for details of the derivation. All analytical results presented in this paper are derived based on this equality.

### Non-pharmaceutical intervention

**Fig. 1:**
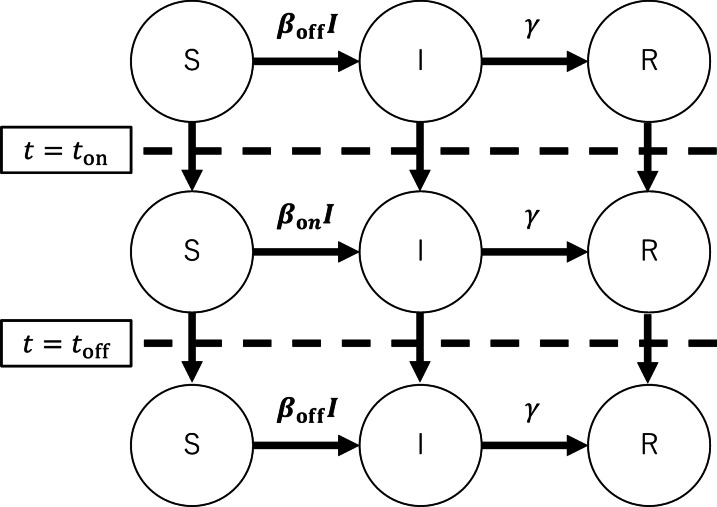
Flowchart of the model. The transmission rate $\beta$ is switched from $\beta _{\mathrm{off}}$ to $\beta _\mathrm{on}(<\beta _{\mathrm{off}})$ during the intervention

In the present framework, an NPI in an isolated population is represented by a change in the transmission rate $\beta$. Let $\beta = \beta _{\mathrm{off}} (>\gamma )$ be the transmission rate without the intervention, and it is switched to $\beta _{\mathrm{on}} (<\beta _\mathrm{off})$ when the intervention starts at $t=t_{\mathrm{on}}$, and restored to $\beta _{\mathrm{off}}$ at $t=t_{\mathrm{off}}=t_{\mathrm{on}}+\Delta t$. See Fig. 1 for the schematic diagram of the setting. Let the corresponding basic reproduction numbers be $R_{0,\mathrm{off}}=\beta _{\mathrm{off}}/\gamma$ and $R_{0,\mathrm{on}}=\beta _{\mathrm{on}}/\gamma$, respectively. Here, $\Delta t$ denotes the duration of the intervention. In this paper, we study the effect of one-shot intervention, one of the simplest implementation schemes, where the intervention is implemented only once. The mathematical methods employed in this study can be applied to more complex cases, for example, where multiple interventions are implemented intermittently. If the fraction of susceptible individuals remains large enough and the herd immunity is not achieved after the intervention, a second wave occurs. It is thus necessary to consider this second wave to evaluate the effect of the intervention.

The one-shot intervention setting has been numerically studied by Bootsma and Ferguson [26], showing that the final size, which depends on the timing $t_{\mathrm{on}}$ and $t_{\mathrm{off}}$, can be smaller for weaker intervention with larger $R_{0,\mathrm{on}}$. Di Lauro et al. [28] numerically studied the dependence of the final size and the peak fraction of infected individuals on the timing of the intervention, where the intervention was assumed to start when the fraction of infected and recovered individuals exceeds a threshold value. In particular, they concluded that the onset timing should be chosen so that the two peaks during and after the intervention are comparable. Morris et al. [29] showed that the maximum fraction of infected individuals with one-shot interventions can approach that achieved by the optimal intervention, which requires an unrealistic intervention, such as $R_{0,\mathrm{on}}=0$. Sadeghi et al. [30] also suggested the existence of the optimal timing of the intervention based on discussion using a linearized equation and numerical simulation.

## Analysis

**Fig. 2:**
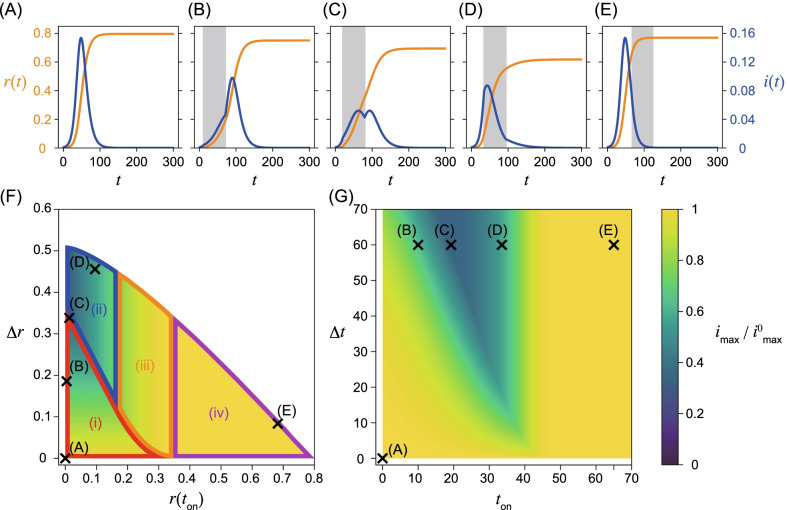
Weak intervention with large $R_{0,\mathrm{on}}=1.4>1$. Parameters are $\beta _{\mathrm{off}}=2/7 \ \mathrm{days}^{-1}$ and $\gamma =1/7 \ \mathrm{days}^{-1}$. **A** Time series of the fraction of infected and removed individuals, *i*(*t*) in blue and *r*(*t*) in orange, without the intervention. Time series with the intervention with $\beta _{\mathrm{on}}=1.4/7 \ \mathrm{days}^{-1}$ and the intervention duration, $\Delta t=60$ days, with onset times **B** $t_{\mathrm{on}}= 10$ days, **C** $t_{\mathrm{on}}= 19.2$ days, **D** $t_{\mathrm{on}}=33.4$ days, and **E** $t_{\mathrm{on}}=65$ days, are depicted. The intervention is implemented for $t_{\mathrm{on}}\le t \le t_{\mathrm{off}}=t_{\mathrm{on}}+\Delta t$ and are represented by grey intervals in these panels. Conditions for the peaks of the fraction of infected individuals are given by $s(t)=1/R_{0,\mathrm{off}}$, when the intervention is not implemented, and by $s(t)=1/R_{0,\mathrm{on}}$ during intervention. The final size of the outbreak for each case is represented by $r(\infty )$. **F** and **G** represent the maximum fraction of infected individuals $i_{\mathrm{max}}$ normalized by that without the intervention $i_\mathrm{max}^0$, plotted in terms of $r(t_{\mathrm{on}})$ and $\Delta r = r(t_{\mathrm{off}})-r(t_{\mathrm{on}})$, and $t_{\mathrm{on}}$ and $\Delta t$, respectively. Symbols **A**–**E** in **F** and **G** denote the intervention timings of the time series of the corresponding in **A**–**E**. Symbols (i)-(iv) in **F** denote the timing that the maximum infected fraction is observed, as described in the main text. The boundaries between regions shown in **F** are obtained analytically. See the main text for the details

**Fig. 3:**
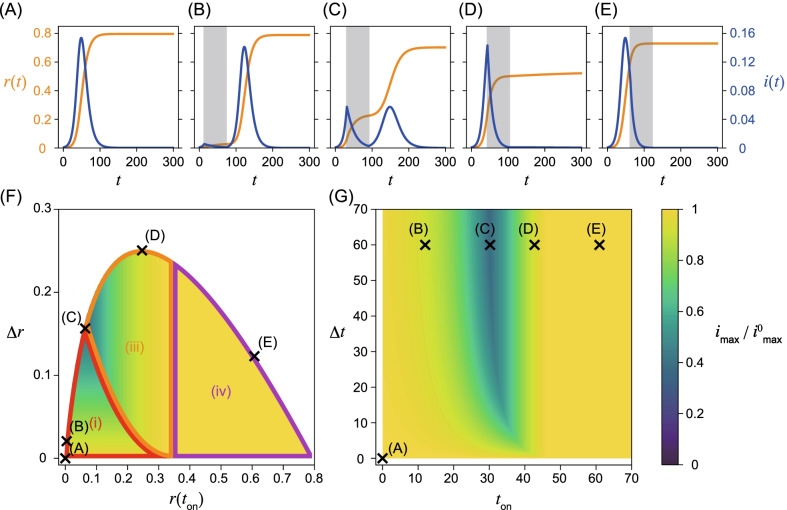
Strong intervention with small $R_{0,\mathrm{on}}=0.7<1$. Parameters are $\beta _{\mathrm{off}}=2/7 \ \mathrm{days}^{-1}$ and $\gamma =1/7 \ \mathrm{days}^{-1}$. **A** Time series of the fraction of infected and removed individuals, *i*(*t*) in blue and *r*(*t*) in orange, without intervention. Note that this time series is identical to that presented in Fig. 2(A). Time series with intervention with $\beta _{\mathrm{on}}=0.7/7 \ \mathrm{days}^{-1}$ and the intervention duration $\Delta t=60$ days with onset times **B** $t_{\mathrm{on}}=12$ days, **C** $t_{\mathrm{on}}=30.2$ days, **D** $t_{\mathrm{on}}=42.7$ days, and **E** $t_{\mathrm{on}}=61$ days. The intervention is implemented for $t_{\mathrm{on}}\le t \le t_\mathrm{off}=t_{\mathrm{on}}+\Delta t$ and are represented by the grey intervals. Symbols (i), (ii), and (iv) in **F** denote the times at which the maximum infected fraction is observed. Region (ii) is not observed in this case. Notations of other symbols are the same as those in Fig. 2

Equations (1) and (2) were numerically integrated using open-source numerical solvers in Python. The codes and datasets generated by them are available in the repository [31]. The time step was set to 0.001. The nonintervention transmission rate and the recovery rate are fixed as $\beta _{\mathrm{off}}=2/7 \ \mathrm{days}^{-1}$ and $\gamma =1/7 \ \mathrm{day}^{-1}$, that is, $R_{0,\mathrm{off}}=2$. The initial condition $s(0)=1-\epsilon$, $i(0)=\epsilon$, and $r(0)=0$, where $\epsilon =0.001$ is employed in this study. As discussed in detail below, the system behaves qualitatively differently for different values of $R_{0,\mathrm{on}}$. Here, we primarily focus on the cases for $R_{0,\mathrm{on}}=1.4$ (Fig. 2) and $R_{0,\mathrm{on}}=0.7$ (Fig. 3), corresponding to relatively weaker and stronger intervention intensities, respectively. Note that $R_{0,\mathrm{on}}>1$ in the former case implies that the infection may spread even in the presence of the intervention. The time series without the intervention is shown in Figs. 2A and 3A. They are identical, because $R_{0,\mathrm{on}}$ does not affect the dynamics without the intervention. Figures 2B–E and 3B–E show the time series with an intervention with a constant intervention duration $\Delta t=60$ days. The second wave can occur if the herd immunity is not achieved when the intervention ends (Figs. 2C and 3B, C). Note that these time series approximate the time evolution observed in an agent-based model on a random network with the corresponding parameter values and initial conditions. The comparisons of the time series are given in Additional file 1: Section S2.

### Maximum fraction of infected individuals

In addition to the numerically obtained time series, we analytically show that peaks of the fraction of infected individuals can appear at the following four timings by applying Eq. (4): after the intervention, during the intervention, at the onset of the intervention, and before the intervention. Note that there can be two peaks of the fraction of infected individuals if a second wave occurs. In such a case, the peak with a larger fraction gives the global maximum. Let us describe the four cases with respect to the timing of the maximum in detail.

(i) The maximum appears after the intervention (Figs. 2B, C, 3B, C). If the intervention ends before achieving herd immunity, a peak is observed during the second wave after the intervention. The fraction of infected individuals for this peak gives the global maximum if it is higher than the first peak before or during the intervention.
(ii) The maximum appears during the intervention (Fig. 2D). If the effective reproduction number $R_t$ declines and crosses unity during the intervention, there is a peak in this timing. The condition $R_{0, \mathrm{on}}>1$ is necessary for the existence of this peak, because the effective reproduction number has to be larger than unity at the onset of the intervention. Therefore, this peak does not appear for $R_{0,\mathrm{on}}<1$. This peak is the global maximum if it is larger than the second peak. The peak also appears at this timing in Fig. 2C, but the second peak is slightly higher than this peak.
(iii) The maximum appears at the onset of the intervention at $t=t_{\mathrm{on}}$ (Fig. 3D). If $R_{t_\mathrm{on}}<1$ holds when the intervention starts, the fraction of infected individuals decreases during the intervention, and we observe a local peak at $t=t_{\mathrm{on}}$. There may be another peak if herd immunity is not achieved during the intervention. If the fraction of infected individuals at the second peak is less than at this peak, the timing of the global maximum is given at $t_{\mathrm{on}}$. Note that this region also appears for $R_{0,\mathrm{on}}>1$, although the time series is not shown in Fig. 2B–E.
(iv) The maximum appears before the onset of the intervention (Figs. 2E and 3E). The fraction of infected individuals reaches its maximum before the intervention. This case implies that the intervention starts too late and fails to mitigate outbreaks in terms of the maximum fraction of infected individuals.

The fraction of infected individuals at the peaks can be calculated analytically. Conditions for the peaks are given in terms of the fraction of susceptible individuals as $s(t)=1/R_{0,\mathrm{off}}=\gamma /\beta _{\mathrm{off}}$ without the intervention and $s(t)=1/R_{0,\mathrm{on}}=\gamma /\beta _{\mathrm{on}}$ with the intervention. The maximum fraction of infected individuals for cases (ii), (iii), and (iv) does not depend on the fraction of removed individuals at the offset of the intervention $r(t_{\mathrm{off}})$, because these peaks appear before $t_{\mathrm{off}}$. For case (i), the maximum fraction of infected individuals $i_{\mathrm{max}}$ is given as

5 $$\begin{aligned} i_{\mathrm{max}}= & {} 1 - \left( 1- \frac{R_{0,\mathrm{on}}}{R_{0,\mathrm{ off}}}\right) \Delta r - \frac{1}{R_{0,\mathrm{off}}} \bigg [ 1 + \log \left( R_{0,\mathrm{ off}} \right) \bigg ], \end{aligned}$$

which depends on $\Delta r:= r(t_{\mathrm{off}})-r(t_{\mathrm{on}})$, the difference in the fraction of removed individuals between the onset and offset of the intervention. See Additional file 1: Section S3 for the explicit form of $i_{\mathrm{max}}$ for all cases and its derivation.

The boundaries between regions (i) and (ii), (i) and (iii), (ii) and (iii), and (iii) and (iv) can be obtained analytically with respect to $r(t_{\mathrm{on}})$ and $\Delta r$ (Figs. 2F and 3F). On the boundaries, the fraction of infected individuals at two peaks is comparable (Figs. 2C and 3C). See Additional file 1: Section S4 for details of the derivation.

### Final size with intervention

The final size also reflects the effect of the intervention. The final size of removed individuals $r(\infty )$ with the intervention is obtained by solving the equation

6 $$\begin{aligned} r(\infty )= & {} 1 - \exp \bigg [\big (R_{0,\mathrm{off}}-R_{0,\mathrm{on}}\big ) \Delta r \bigg ] \exp \bigg [- R_{0,\mathrm{off}} \ r(\infty ) \bigg ], \end{aligned}$$

in a self-consistent manner [32]. Specifically, as $r(\infty )$ appears on both sides, this equation can be solved numerically or using the Lambert *W* function, except for some special cases. This equation implies that the final size depends on $\Delta r = r(t_{\mathrm{off}})-r(t_{\mathrm{on}})$. Another important implication of this equation is that $r(t_{\mathrm{off}})$ has the upper bound ${\tilde{r}}$ depending on $r(t_{\mathrm{on}})$, which is given by

7 $$\begin{aligned} {\tilde{r}}(r(t_{\mathrm{on}}))= & {} 1 - \exp \bigg [-(R_{0,\mathrm{off}} - R_{0,\mathrm{on}}) r(t_{\mathrm{on}})\bigg ] \exp \bigg [-R_{0,\mathrm{on}} \ {\tilde{r}}\bigg ]. \end{aligned}$$

See Additional file 1: Section S5 for the details of the derivation. The final state represented by Eq. (6) is the equilibrium in the presence of the intervention. One can show that the Jacobian matrix of the linearized equation for this final state has zero eigenvalue, which implies that this equilibrium is neutrally stable See Additional file 1: Section S6 for the derivation of the detailed discussion.

Using this equality, one can show that the final size in the presence of the intervention is always smaller than that without the intervention. For

8 $$\begin{aligned} R_{0,\mathrm{on}}< & {} \frac{R_{0,\mathrm{off}} }{R_{0,\mathrm{off}}-1} \log \left( R_{0,\mathrm{off}} \right) , \end{aligned}$$

one can achieve $r(\infty )\approx 1-1/R_{0,\mathrm{off}}$ by setting $t_{\mathrm{on}}$ properly, which is the smallest prevalence to achieve herd immunity, with an intervention duration $\Delta t$ that is large enough [32]. See Supplementary Additional file 1: Section S6 for details of the derivation. Numerical results regarding the final size are summarized in Additional file 1: Section S7.

## Results

We report the numerical and analytical results, when the reproduction number under the intervention $R_{0,\mathrm{on}}$ is large (Fig. 2) and small (Fig. 3), showing qualitatively different behaviors.

In Figs. 2F, G, 3F, G, the dependence of the maximum fraction of infected individuals on the timing of the intervention is plotted. Here, $i_{\mathrm{max}}$ is normalized by that in the absence of the intervention $i_{\mathrm{max}}^{0}$ (Figs. 2A and 3A). As the maximum infected fraction drops in the presence of the intervention, $i_{\mathrm{max}}/i_{\mathrm{max}}^0$ is less than unity and quantifies the effectiveness of the intervention in terms of the maximum fraction of infected individuals. As this ratio decreases, the intervention shows more success in reducing the maximum fraction.

It is difficult to obtain the time series of the SIR model analytically without any approximations, for example, linearization, or the method presented in [6]. Therefore, $i_{\mathrm{max}}$ is numerically computed with different onset and offset times for the intervention, $t_{\mathrm{on}}$ and $t_{\mathrm{off}}$ (Figs. 2G and 3G). However, $i_{\mathrm{max}}$ can be analytically calculated with respect to the fraction of the recovered individuals at the onset, $r(t_\mathrm{on})$, and the offset, $r(t_{\mathrm{off}})$, of the intervention (Figs. 2F and 3F). Note that there exists a one-to-one correspondence between $\{t_\mathrm{on},t_{\mathrm{off}} \}$ (panels (F)) and $\{ r(t_{\mathrm{on}}), r(t_\mathrm{off}) \}$ (panels (G)) in Figs. 2 and 3.

### Weak intervention (large $R_{0,\mathrm{on}}=1.4$)

In this case, the reproduction number is larger than unity even in the presence of the intervention. Therefore, the fraction of infected individuals may increase during the intervention period. The peaks of infected individuals can be observed during the intervention, and the maximum infected fraction can appear at any of the four timings (i)–(iv) classified above. Figures 2B–E show that $i_{\mathrm{max}}$ is minimized in the intermediate onset time $t_{\mathrm{on}}$ near (C), where the peaks during and after the intervention are comparable. This non-monotonic dependence on $t_{\mathrm{on}}$ is clearly visualized in Fig. 2G. The peak of infected individuals during the intervention is smaller than that without intervention. This intervention mitigates the second wave.

As shown in Eq. (5) and Fig. 2F, the maximum fraction of infected individuals is linear in $\Delta r$ if the peak of the second wave is the maximum, that is, case (i). This is verified by the fact that the contours lie horizontally in case (i). To clarify this point, the contours are explicitly shown in Additional file 1: Section S8. If the fraction of the infected individuals reaches the maximum during or at the onset of the intervention (cases (ii) and (iii), respectively), the maximum fraction depends only on $t_{\mathrm{on}}$, which has one-to-one correspondence to $r(t_{\mathrm{on}})$. This is verified in Fig. 2F, G, and Additional file 1: Section S8. If the maximum appears before the intervention, that is, case (iv), the maximum is independent of both $t_{\mathrm{on}}$ and $t_{\mathrm{off}}$.

Onset and offset times for the intervention with constant $\Delta t$ corresponding to Figs. 2A–E are plotted in Figs. 2F, G. As suggested by the time series, the maximum infected fraction is smallest in the intermediate intervention onset $t_{\mathrm{on}}$, near (C). It is clear from panel (F) that this point is located close to the boundary between cases (i) and (ii), where peaks during and after the intervention are comparable. In case (ii), the maximum does not depend on $\Delta r$, which implies that a longer intervention does not reduce the maximum. Note that the relationship between $(t_\mathrm{on},t_{\mathrm{off}})$ and $(r(t_{\mathrm{on}}),r(t_{\mathrm{off}}))$ is non-monotonous.

### Strong intervention (small $R_{0,\mathrm{on}}=0.7$)

When the transmission rate is small during the intervention, the maximum infected fraction is minimized in the intermediate starting time of the intervention $t_{\mathrm{on}}$, namely, early implementation of the intervention does not necessarily minimize the infected fraction (Figs. 3F, G). This result is intuitively understood as follows. If the intervention starts too early, the infection does not spread because of the small intervention transmission rate. Therefore, the second wave after the intervention is large, thus the early intervention is not effective. If the timing of the intervention is characterized in terms of $r(t_{\mathrm{on}})$ and $r(t_{\mathrm{off}})$ (Fig. 3F), the maximum of the second wave depends only on $\Delta r$. If the maximum fraction is found during the intervention, its value is independent of $t_{\mathrm{off}}$ and $r(t_{\mathrm{off}})$. The peak does not appear during the intervention, that is, case (ii) does not appear because $R_{t,\mathrm{on}}<1$ holds.

### Maximum infected fraction ${\bar{i}}_{\mathrm{max}}$ versus reproduction number under the intervention $R_{0,\mathrm{on}}$

**Fig. 4:**
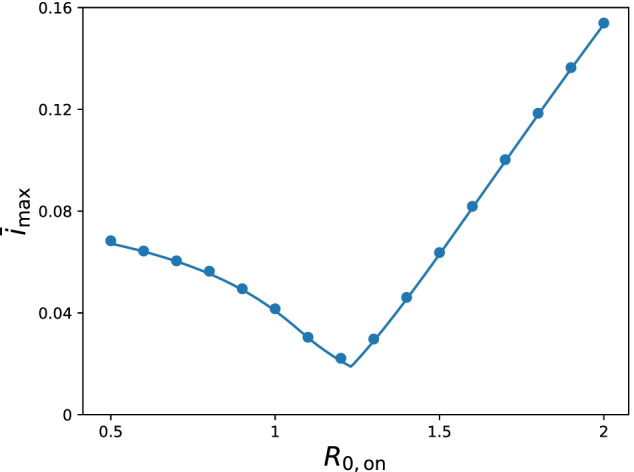
Dependence of ${\bar{i}}_{\mathrm{max}}$, the maximum fraction of infected individuals minimized by choosing the onset and offset timings of intervention, on the intervention reproduction number $R_{0,\mathrm{on}}$. The symbols and solid line represent the numerical and analytical results (Additional file 1: Section S9), respectively. The numerical results verifies the theoretical prediction that ${\bar{i}}_{\mathrm{max}}$ takes the minimum value at $R^*_{0,\mathrm{on}}\approx 1.23$

For each $R_{0,\mathrm{on}}$, there exist onset and offset timings of the intervention that minimize the maximum fraction of infected individuals $i_{\mathrm{max}}$ (Figs. 2F, G and 3F, G). Let this value be ${\bar{i}}_\mathrm{max}(R_{0,\mathrm{on}})$. Figure 4 plots the dependence of ${\bar{i}}_{\mathrm{max}}$ on $R_{0,\mathrm{on}}$. As seen in the figure, it is minimized at a non-trivial intermediate value of $R_{0,\mathrm{on}}=R_{0,\mathrm{on}}^{*}\approx 1.23>1$. This implies that the maximum fraction of infected individuals is minimized for a weak intervention under the one-shot condition. For $R_{0,\mathrm{on}}\ge R_{0,\mathrm{on}}^*$, ${\bar{i}}_{\mathrm{max}}$ is achieved at the boundary between regions (i) and (ii) at $t_{\mathrm{on}}=0$, where the peaks during and after the intervention are comparable (Fig. 2C). For $R_{0,\mathrm{on}}^*\ge R_{0,\mathrm{on}} \ge R_{0,\mathrm{on}}^{(1)}$, the boundary between regions (i) and (ii) with $t_{\mathrm{on}}>0$ gives ${\bar{i}}_{\mathrm{max}}$, where $R_{0,\mathrm{on}}^{(1)}\approx 1.08$ is the parameter value below which region (ii) does not exist. For strong intervention $R_{0,\mathrm{on}}\le R_{0,\mathrm{on}}^{(1)}$, ${\bar{i}}_{\mathrm{max}}$ is found at the boundary between regions (i) and (iii), where the peaks of the onset and after the intervention are comparable (Fig. 3(C)), at $t_{\mathrm{on}}>0$. Conditions for ${\bar{i}}_{\mathrm{max}}$ are analytically derived in all cases, shown by the solid line in Fig. 4. The conditions for ${\bar{i}}_{\mathrm{max}}$ for $R_{0,\mathrm{on}}\ge R_{0,\mathrm{on}}^{(1)}$ can be explicitly solved. The condition for small $R_{0,\mathrm{on}}\le R_{0,\mathrm{on}}^{(1)}$ cannot be solved explicitly, and the parametric equations for ${\bar{i}}_{\mathrm{max}}$ and $R_{0,\mathrm{on}}$ are used to plot the theoretical curve in Fig. 4. See Additional file 1: Section S9 for details of the derivation.

## Intervention strategies

It is possile to optimize the onset and offset timings of the NPI by minimizing an objective function under certain constraints. In the present framework, this can be formulated as an optimization problem in a hybrid nonlinear dynamical system. The optimal intervention strategy depends on the objective function. Here, we discuss the following two simple scenarios to minimize $i_{\mathrm{max}}$. In general, more complex objective functions can be used for optimization. The following discussion may provide some intuition for considering such cases, taking into account the second wave.

### Minimizing $i_{\mathrm{max}}$ with a constraint in the intervention duration

Let us start with a case where the intervention duration $\Delta t$ is less than a certain value. As $i_{\mathrm{max}}$ is monotonically decreasing in $\Delta t$ for a fixed $t_{\mathrm{on}}$, we can assume that $\Delta t$ is a constant, and $t_{\mathrm{on}}$ is varied. This case has been studied in Figs. 2B–E and 3B–E. This scheme fixes the intervention duration, so it is easier to anticipate the economic impact of the intervention, which depends on the duration of the intervention, than in the next scenario based on the fraction of recovered individuals.

As discussed above, $i_{\mathrm{max}}$ is minimized in the intermediate onset time $t_{\mathrm{on}}$, when the first peak during the intervention and the second peak after the intervention are comparable. For larger $R_{0,\mathrm{on}}$, $t_{\mathrm{on}}$ giving the minimum $i_{\mathrm{max}}$ converges to zero for large $\Delta t$ (Fig. 2G), but converges to non-zero for large $\Delta t$ for smaller $R_{0,\mathrm{on}}$ (Fig. 3(G)). These results are understood as follows: for a strong intervention with small $R_{0,\mathrm{on}}$, the intervention immediately suppresses the fraction of infected individuals. Therefore, the early onset of the intervention prevents more individuals from achieving immunity during the intervention and eventually increases the maximum fraction of infected individuals.

### Minimizing intervention duration with a constraint in $i_{\mathrm{max}}$

Another possible constraint is to minimize the intervention duration $\Delta t$, keeping the maximum fraction of infected individuals $i_{\mathrm{max}}$ constant. This corresponds to choosing $t_{\mathrm{on}}$ along a contour of $i_{\mathrm{max}}$, which prevents the overcapacity of medical support. In Figs. 2G and 3G, $\Delta t$ is minimized for an intermediate $t_{\mathrm{on}}$. Namely, a contour that crosses point (C) ($\Delta t=60$) reaches $\Delta t\approx 20$ if the onset time is later than $t_{\mathrm{on}}=19.2$ (Fig. 2(C)). As we have already discussed, the maximum fraction depends on $\Delta r$ in region (i). It takes a shorter time to achieve the same $\Delta r$ if the intervention starts later in this case. As the maximum fraction of infected individuals depends on $r(t_{\mathrm{on}})$ but is independent of $\Delta r$ in cases (ii) and (iii), the optimal $r(t_{\mathrm{on}})$ is determined by the boundary between regions (i) and (ii) or that between (i) and (iii) in Figs. 2F and 3F. Evidently this timing is an intermediate value of $t_{\mathrm{on}}> 0$. It may be easier to set a plan for the intervention with respect to $r(t_{\mathrm{on}})$ and $r(t_{\mathrm{off}})$, rather than the timing $t_\mathrm{on}$ and $t_{\mathrm{off}}$.

Similar optimization problems can be considered using the final size as the objective functions. These problems are discussed in Additional file 1: Section S10.

## Conclusion

In the present study, in consideration of the actual COVID-19 situation, we studied the situation in which the reproduction number of an infectious disease is temporarily reduced by implementing an NPI once during the epidemic, using a simple mathematical model. The results provide theoretical implications as to how strong NPIs should be introduced during an epidemic of an emerging infectious disease. If the effective reproduction number during the intervention is too small, the fraction of infected individuals at the peak in the second wave may be higher than the first peak. It was also shown numerically and analytically that the fraction of infected individuals can also increase if the intervention is started too early. The upper limit of medical capacity is an essential practical constraint. In particular, for infectious diseases such as COVID-19, which is too emergent to expect effective treatments, it is more important to avoid exhausting the medical system. This study suggests that it will be necessary to be alert for a larger second wave that may occur after strong intervention in such cases.

We analytically derived the peak fractions of infected individuals in the SIR model with the one-shot intervention. These analytical results suggest that the peak fractions can be smaller with non-trivial intervention timings. The maximum fraction is smallest for $R_{0,\mathrm{on}}>1$, that is, the intervention reproduction number is not in the disease-free regime. In the literature, Bootsma and Ferguson [26] showed that the final size can be minimized for non-trivial intervention timing and $R_{0,\mathrm{on}}$ numerically. Di Lauro et al. [28] numerically showed that the peak fraction of infected individuals also depends on the timing of the intervention. We obtained analytical expressions for these quantities in this study. The analytical results for the final size are presented in Additional file 1: Section S6. It should be noted that the formulae for the peak fraction depend on the timing of the peak, resulting in various cases compared with the final size. Some analytical results are available for this system. Sadeghi et al. [30] explained these non-trivial effects based on solutions of the linearized equation, which exponentially grows and decays without and with the intervention, respectively. Linearization is one of the simplest approximations and is applicable in this case; in particular, this approximation is useful in discussions regarding timing. Conversely, linearization cannot be used to discuss the important case where $R_{0,\mathrm{on}}>1$, as the linearized equation cannot explain the declining number of infected individuals. The proposed method in this study provides a unified framework, including the cases where linearization is not feasible. Morris et al. derived [29] an equation for the peaks of the fraction of infected individuals in terms of $s(t_{\mathrm{on}})$, $s(t_{\mathrm{off}})$, $i(t_{\mathrm{on}})$, and $i(t_{\mathrm{off}})$. In this work, we further show the peak fractions in terms of the two parameters $r(t_{\mathrm{on}})$ and $r(t_{\mathrm{off}})$ for a general initial condition.

Although concrete measures and guidelines for COVID-19 are required, it is emphasized again that the results in this study were derived using a simplified model with many assumptions. First, the results presented in this study are based on the simplified SIR model, which takes into account neither the realistic pathology of COVID-19 nor societal response. This model assumes uniform and random contact within a group and does not consider interactions between different subgroups in the population. Recovered patients are assumed to have complete immunity in this model. These assumptions are not applicable to COVID-19, where there are still many unclear factors regarding heterogeneity in contact networks and the immune response of patients after recovery. It has been shown that heterogeneous contacts can affect the infection dynamics [33]. The presented method could be applied in such a case, if the mean field approximation describes the epidemic process well. If a society develops other public health measures during the intervention, the basic reproduction numbers before and after the NPI may be different. Next, this study is a one-shot intervention model, in which the transmission coefficient returns to the original value after a single intervention. Practically, each re-pandemic requires multiple intermittent interventions [34], making the intervention process much more complex [35]. Change in the parameter $\beta$ between $\beta _{\mathrm{off}}$ and $\beta _{\mathrm{on}}$ occurs discontinuously in the present model. However, it should take a finite time in realistic case reflecting the time for responses of people to an NPI [36]. The finite time interval for the parameter change gives a correction to the results presented here. Detailed discussions are given in Additional file 1: Section S11. Furthermore, the model is based on a deterministic dynamical system of an isolated population. Therefore, important NPI measures such as border control cannot be estimated in the present framework. The deterministic nature of the model assumes that the infectious disease cannot be eradicated, as the number of infected individuals remains non-zero for a finite time. If the population is small enough and no imported cases are assumed, strong and early intervention, which is not necessarily recommended in this study, may eradicate the disease, and a second wave does not occur. Strong intervention would be necessary in other cases, for example, when the number of infected patients approaches the capacity of the medical system. These complex situations may be analyzed in detail by extending the methods presented in this study to e.g., stochastic epidemic models [37], which may lead to different conclusions from those reached in this study. Another important situation would be cases where the endemic state exists [38, 39]. Such systems behave qualitatively differently, e.g., existence of the Lyapunov function may lead to the global stability of the steady state. It would be important to discuss an effective intervention strategies to such systems.

It is also important to consider dynamics using a different criterion, such as the fraction of new cases, as a trigger of an NPI. Here, let us briefly discuss the possibility to use *i*(*t*) for onset/offset criteria for the intervention. If we can map *i*(*t*) to *r*(*t*), then we can apply the present framework. The conservation of the total population $i(t) = 1 - r(t) - s(t) = 1 - r(t) - \exp (- R_{0,\mathrm{off}} \, r(t))$ enables to determine *i*(*t*) from *r*(*t*) at the onset of the intervention. However, there can be multiple *r*(*t*) values corresponding to a given *i*(*t*). Therefore, if we specify the branch of *r*(*t*), we can apply the present framework using *i*(*t*) as the trigger of the intervention. The detailed formalization would be an imporntant future work.

In this study, the effect of NPIs was modeled as a hybrid dynamical system, which may further enable us to approach more refined models in future investigations. The influence of NPIs with respect to political decisions and behavioral changes of people can be expressed more accurately by introducing a hybrid dynamical system. In recent years, dynamical systems theory and control theory have been developed, and phenomena specific to hybrid systems such as Zeno solutions and sliding motions have been discussed [18–22]. Some studies, such as [40] and [41], have proposed control of infectious diseases using sliding mode control. The optimal policy under NPIs can be discussed by modeling the effect of economic damages associated with execution and then minimizing the cost function. Discrete changes in parameters such as the transmission coefficient in NPI implementation are strongly linked to the intensity of measures, suppression of economic activity, and changes in human mobility. For example, the correlation between the decrease in human mobility with NPIs and the effective reproduction number for the COVID-19 pandemic has been studied [42, 43]. Such studies can contribute to modeling the costs of NPIs. The mathematical model of the epidemic suppression effect can be constructed using a hybrid dynamical system, taking into account the negative socioeconomic impact of NPIs.

## Supplementary Information

**Additional file 1.** Detailed discussions carried out in the main text are given.

## Data Availability

The datasets generated and analysed during the current study are available in the repository [31].
