# Supplementary material for: Analytical estimation of maximum fraction of infected individuals with one-shot non-pharmaceutical intervention in a hybrid epidemic model
Source: BMC Infect Dis. 2022 Jun 1;22:512. doi: 10.1186/s12879-022-07403-5 (PMC9157046; doi:10.1186/s12879-022-07403-5)
Supplement: Supplementary file 1 — Additional file 1. Detailed discussions carried out in the main text are given. [file 12879_2022_7403_MOESM1_ESM.pdf]

# Supplementary materials: Analytical estimation of maximum fraction of infected individuals with one-shot non-pharmaceutical intervention in a hybrid epidemic model

## S1 Derivation of Eq. (4) [1–4]

We want to derive Eq. (4) in the main text from the evolution equation of the SIR model

$$\frac{ds}{dt} = -\beta si, \tag{S1}$$
$$\frac{di}{dt} = \beta si - \gamma i, \tag{S2}$$
$$\frac{dr}{dt} = \gamma i. \tag{S3}$$

We integrate Eq. (S1) from time $t_0$ to $t_1$:

$$s(t_1) = s(t_0)\exp\left[-\beta\int_{t_0}^{t_1} i(t')dt'\right]. \tag{S4}$$

The integral on the right-hand side can be replaced with the integration of Eq. (S3),

$$r(t_1) - r(t_0) = \gamma\int_{t_0}^{t_1} i(t')dt'. \tag{S5}$$

Then, we obtain

$$s(t_1) = s(t_0)\exp\left\{-\frac{\beta}{\gamma}[r(t_1) - r(t_0)]\right\}, \tag{S6}$$

which is Eq. (4) in the main text. We apply this equation to various $t_0$ and $t_1$.

## S2 Stochastic simulations

To verify that the current framework can describe the dynamics of the epidemic spreading based on the agent-based model, we performed the stochastic simulation of the agent-based model on a random network. In the simulation, we used the python package provided by Kiss et al. [5]. Figure S1 shows the comparison between the solutions of the SIR model and the agent-based simulations, corresponding to Figs. 2 and 3 in the main text. As shown in the figure, the solutions of the differential equation approximate well the time series of the agent-based model, suggesting the applicability of the present framework in the case of homogeneously mixed agent-based models.

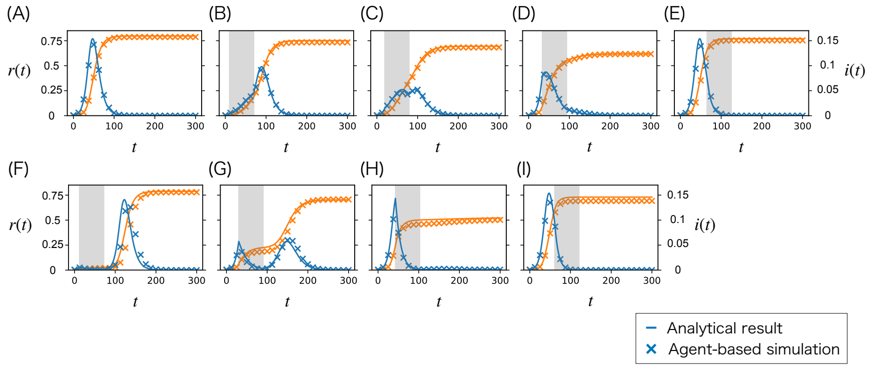


**Figure S 1** Comparison between the solutions of the SIR model (Eqs. (1) and (2) in the main text) and time evolution of the agent-based simulation on a random network. The blue and orange solid lines denote time series of $i(t)$ and $r(t)$ in the SIR model corresponding to Figs. 2(A)-(E) in the main text for panels (A)-(E), and Figs. 3(B)-(E) in the main text for panels (F)-(I). The symbols of the blue and orange crosses denote the time series of the fractions of infected and removed agents in the agent-based simulation averaged over 10 iterations. Paramter values in the agent-based simulation are $\beta_{\mathrm{off}} = 2/7\ \mathrm{days}^{-1}$, $\gamma = 1/7\ \mathrm{days}^{-1}$, fraction of initially infected agents $\epsilon = 0.001$, and duration of the intervention $\Delta t = 60$ days. Values of $\beta_{\mathrm{on}}$ are $1.4/7\ \mathrm{days}^{-1}$ for (B)-(E) and $= 0.7/7\ \mathrm{days}^{-1}$ for (F)-(I). These parameter values are common to those in Figs. 2 and 3 in the main text. Parameters specific to the agent-based model are number of nodes $N = 100,000$, and average degree of the random network $\langle k \rangle = 100$. Grey backgrounds in (B)-(I) in the figures denote the intervention periods.

## S3 Analytical expressions for the maximum fraction of infected individuals

As described in the main text, there are four possible timings where the maximum fraction of infected individuals $i_{\mathrm{max}}$ is observed. We summarize the analytical forms of the maximum fraction in all cases with respect to the fractions of removed individuals at the onset $r(t_{\mathrm{on}})$ and the offset $r(t_{\mathrm{off}})$ of the intervention, that is, we assume that the timing of the onset and offset of the intervention is determined by the fraction of removed individuals.

- **(i) The maximum appears after the intervention:**

$$i_{\mathrm{max}} = 1 - \left(1 - \frac{R_{0,\mathrm{on}}}{R_{0,\mathrm{off}}}\right) \Delta r - \frac{1}{R_{0,\mathrm{off}}} \left[1 + \log\left(R_{0,\mathrm{off}}\right)\right], \tag{S7}$$

where $\Delta r = r(t_{\mathrm{off}}) - r(t_{\mathrm{on}})$.

- **(ii) The maximum appears during the intervention:**

$$i_{\mathrm{max}} = 1 + \left(\frac{R_{0,\mathrm{off}}}{R_{0,\mathrm{on}}} - 1\right) r(t_{\mathrm{on}}) - \frac{1}{R_{0,\mathrm{on}}} \left[1 + \log\left(R_{0,\mathrm{on}}\right)\right]. \tag{S8}$$

- **(iii) The maximum appears at the onset of the intervention:**

$$i_{\mathrm{max}} = 1 - \exp\left[-R_{0,\mathrm{off}}\ r(t_{\mathrm{on}})\right] - r(t_{\mathrm{on}}). \tag{S9}$$

- **(iv) The maximum appears before the onset of the intervention:**

$$i_{\mathrm{max}} = 1 - \frac{1}{R_{0,\mathrm{off}}} \left[1 + \log\left(R_{0,\mathrm{off}}\right)\right]. \tag{S10}$$

Derivation

Here, we derive Eqs. (S7)–(S10). The conditions for the fraction of infected individuals to be the local peak are given by $s(t) = \gamma/\beta_{\rm off} = 1/R_{0,\rm off}$ without the intervention and $s(t) = \gamma/\beta_{\rm on} = 1/R_{0,\rm on}$ with the intervention.

First, let us apply Eq. (S6) until the onset time of the intervention, with $\beta = \beta_{\rm off}$, $t_1 = t_{\rm on}$, and $t_0 = 0$. Using the initial condition $s(0) = 1 - \epsilon$, $i(0) = \epsilon$, $r(0) = 0$, we obtain

$$s(t_{\rm on}) = (1-\epsilon)\exp\left[-\frac{\beta_{\rm off}}{\gamma} r(t_{\rm on})\right]. \qquad \text{(S11)}$$

In the following, we take the limit $\epsilon \to 0$.

Substituting Eq. (S11) into Eq. (S6), one can derive the fraction of susceptible and removed individuals during and after the intervention.

*(i) Maximum fraction after the intervention*

We derive the fraction of infected individuals after the intervention. Let $t_p^{\rm after} > t_{\rm off}$ be the time at which the infected fraction reaches the peak after the intervention. The relationship between $s(t_{\rm off})$ and $r(t_{\rm off})$ is derived by substituting Eq. (S11) with $\epsilon \to 0$ into Eq. (S6) with $t_0 = t_{\rm on}$ and $t_1 = t_{\rm off}$ as follows:

$$s(t_{\rm off}) = \exp\left[-\frac{\beta_{\rm on}}{\gamma} r(t_{\rm off})\right] \exp\left[-\frac{\beta_{\rm off}-\beta_{\rm on}}{\gamma} r(t_{\rm on})\right]. \qquad \text{(S12)}$$

Then, by applying Eq. (S6) setting $t_0 = t_{\rm off}$ and $t_1 = t_p^{\rm after}$, we have

$$s(t_p^{\rm after}) = \exp\left[-\frac{\beta_{\rm off}}{\gamma} r(t_p^{\rm after})\right] \exp\left\{\frac{\beta_{\rm off}-\beta_{\rm on}}{\gamma}\left[r(t_{\rm off}) - r(t_{\rm on})\right]\right\}. \qquad \text{(S13)}$$

The condition for the peak after the intervention is $s(t_p^{\rm after}) = \gamma/\beta_{\rm off}$. Substituting this condition into Eq. (S13), the fractions of removed and infected individuals are given by:

$$\begin{aligned} r(t_p^{\rm after}) &= \frac{\beta_{\rm off}-\beta_{\rm on}}{\beta_{\rm off}}[r(t_{\rm off}) - r(t_{\rm on})] + \frac{\gamma}{\beta_{\rm off}}\log\left(\frac{\beta_{\rm off}}{\gamma}\right), & \text{(S14)} \\ i(t_p^{\rm after}) &= 1 - s(t_p^{\rm after}) - r(t_p^{\rm after}) & \text{(S15)} \\ &= 1 - \frac{\beta_{\rm off}-\beta_{\rm on}}{\beta_{\rm off}}[r(t_{\rm off}) - r(t_{\rm on})] - \frac{\gamma}{\beta_{\rm off}}\left[1 + \log\left(\frac{\beta_{\rm off}}{\gamma}\right)\right], & \text{(S16)} \end{aligned}$$

which is equivalent to Eq. (S7) with $R_{0,\rm off} = \beta_{\rm off}/\gamma$ and $R_{0,\rm on} = \beta_{\rm on}/\gamma$. Equation (S16) gives $i_{\rm max}$ if the maximum appears after the intervention. Note that the fraction of infected individuals depends only on the onset and offset timings as $r(t_{\rm off}) - r(t_{\rm on})$. The later the onset or sooner the offset is, the higher the peak is.

*(ii) Maximum fraction during the intervention*

If the effective reproduction number at the onset of the intervention is greater than unity, $\beta_{\rm on} s(t_{\rm on})/\gamma > 1$, and that at the offset is less than unity, $\beta_{\rm on} s(t_{\rm off})/\gamma < 1$, then there is a peak during the intervention. Let $t_p^{\rm during}$ be the time at which

the infected fraction reaches the peak, where $t_{\rm on} < t_p^{\rm during} < t_{\rm off}$. We obtain the relationship between susceptible and removed individuals during the intervention by applying Eq. (S6) with $t_0 = t_{\rm on}$ and $t_1 = t_p^{\rm during}$ and using Eq. (S11) as the relationship between $s(t_{\rm on})$ and $r(t_{\rm on})$:

$$s(t_p^{\rm during}) = \exp\left[-\frac{\beta_{\rm on}}{\gamma} r(t_p^{\rm during})\right] \exp\left[-\frac{\beta_{\rm off}-\beta_{\rm on}}{\gamma} r(t_{\rm on})\right]. \tag{S17}$$

The condition for the peak during the intervention is $s(t_p^{\rm during}) = \gamma/\beta_{\rm on}$. By substituting this condition into Eq. (S17), we obtain

$$r(t_p^{\rm during}) = \frac{\gamma}{\beta_{\rm on}}\left[-\frac{1}{\gamma}(\beta_{\rm off}-\beta_{\rm on})r(t_{\rm on}) + \log\left(\frac{\beta_{\rm on}}{\gamma}\right)\right]. \tag{S18}$$

Then, the fraction of infected individuals at this peak is

$$\begin{aligned} i(t_p^{\rm during}) &= 1 - s(t_p^{\rm during}) - r(t_p^{\rm during}) && \text{(S19)}\\ &= 1 + \frac{\beta_{\rm off}-\beta_{\rm on}}{\beta_{\rm on}} r(t_{\rm on}) - \frac{\gamma}{\beta_{\rm on}}\left[1 + \log\left(\frac{\beta_{\rm on}}{\gamma}\right)\right], && \text{(S20)} \end{aligned}$$

which is equivalent to Eq. (S8). As $\beta_{\rm off} > \beta_{\rm on}$ and $\frac{\beta_{\rm off}-\beta_{\rm on}}{\beta_{\rm on}} > 0$, the fraction of infected individuals at this peak linearly increases compared with the fraction of removed individuals at the onset of the intervention $r(t_{\rm on})$. Equation (S20) is the condition for the maximum if this peak is higher than another peak.

The conditions for the existence of this peak are $\beta_{\rm on} s(t_{\rm on}) > \gamma$ and $\beta_{\rm on} s(t_{\rm off}) < \gamma$, which are interpreted as

$$\begin{aligned} r(t_{\rm on}) &< \frac{\gamma}{\beta_{\rm off}} \log\left(\frac{\beta_{\rm on}}{\gamma}\right), && \text{(S21)}\\ r(t_{\rm off}) &> \frac{\gamma}{\beta_{\rm on}}\left[-\frac{1}{\gamma}(\beta_{\rm off}-\beta_{\rm on})r(t_{\rm on}) + \log\left(\frac{\beta_{\rm on}}{\gamma}\right)\right]. && \text{(S22)} \end{aligned}$$

Therefore, $\beta_{\rm on} > \gamma$ is required for the existence of this peak.

*(iii) Maximum fraction at the onset of the intervention*

At the onset of the intervention, the fraction of infected individuals is given as

$$\begin{aligned} i(t_{\rm on}) &= 1 - s(t_{\rm on}) - r(t_{\rm on}) && \text{(S23)}\\ &= 1 - \exp\left[-\frac{\beta_{\rm off}}{\gamma} r(t_{\rm on})\right] - r(t_{\rm on}), && \text{(S24)} \end{aligned}$$

by substituting Eq. (S11).

This peak appears if the effective reproduction number before the intervention is larger than unity $\beta_{\rm off} s(t_{\rm on}) > \gamma$ and that after the onset of the intervention is less than unity $\beta_{\rm on} s(t_{\rm on}) < \gamma$. These conditions are summarized in terms of $r(t_{\rm on})$ as follows:

$$\max\left\{0, \frac{\gamma}{\beta_{\rm off}} \log\left(\frac{\beta_{\rm on}}{\gamma}\right)\right\} < r(t_{\rm on}) < \frac{\gamma}{\beta_{\rm off}} \log\left(\frac{\beta_{\rm off}}{\gamma}\right), \tag{S25}$$

by substituting the above conditions into Eq. (S11).

*(iv) Maximum fraction before the intervention*
Let $t_1 = t_p^{\text{before}}$ be the time at which the peak appears before the onset of the intervention. We apply Eq. (S6) with $\beta = \beta_{\text{off}}$, $t_0 = 0$, and $t_1 = t_p^{\text{before}}$. Then, we obtain

$$r(t_p^{\text{before}}) = \frac{\gamma}{\beta_{\text{off}}} \log \left[ \frac{1}{s(t_p^{\text{before}})} \right]. \quad \text{(S26)}$$

The peak condition $s(t_p^{\text{before}}) = \gamma/\beta_{\text{off}}$ leads to

$$r(t_p^{\text{before}}) = \frac{\gamma}{\beta_{\text{off}}} \log \left( \frac{\beta_{\text{off}}}{\gamma} \right). \quad \text{(S27)}$$

Substituting this equation into the conservation of total population $i(t) = 1 - s(t) - r(t)$, we obtain

$$i(t_p^{\text{before}}) = 1 - \frac{\gamma}{\beta_{\text{off}}} \left[ 1 + \log \left( \frac{\beta_{\text{off}}}{\gamma} \right) \right]. \quad \text{(S28)}$$

This peak appears for $r(t_{\text{on}}) > \frac{\gamma}{\beta_{\text{off}}} \log \left( \frac{\beta_{\text{off}}}{\gamma} \right)$, meaning a late onset of the intervention.

## S4 Derivation of boundaries between regions of different maxima

As shown in Figs. 2(F) and 3(F) in the main text, the timing giving the maximum fraction of infected individuals switches in the $(r(t_{\text{on}}), \Delta r)$ plane, where $\Delta r = r(t_{\text{off}}) - r(t_{\text{on}})$. These figures suggest possible transitions between regions (i) and (ii), between (i) and (iii), between (ii) and (iii), and between (iii) and (iv) listed above. At the boundaries, the peaks of the different timings are expected to be equal. Based on this idea, we sketch the derivations of the equations for the boundaries between these timings.

### Boundaries between regions (i) and (ii)

In regions where peaks of infected individuals during and after the intervention coexist, the global maximum is given by

$$\begin{aligned} i_{\max} &= \max[i(t_p^{\text{after}}), i(t_p^{\text{during}})] & \text{(S29)} \\ &= \max \left\{ 1 - \frac{\beta_{\text{off}} - \beta_{\text{on}}}{\beta_{\text{off}}} [r(t_{\text{off}}) - r(t_{\text{on}})] - \frac{\gamma}{\beta_{\text{off}}} \left[ 1 + \log \left( \frac{\beta_{\text{off}}}{\gamma} \right) \right], \right. \\ &\quad \left. 1 + \frac{\beta_{\text{off}} - \beta_{\text{on}}}{\beta_{\text{on}}} r(t_{\text{on}}) - \frac{\gamma}{\beta_{\text{on}}} \left[ 1 + \log \left( \frac{\beta_{\text{on}}}{\gamma} \right) \right] \right\}. & \text{(S30)} \end{aligned}$$

There occurs a transition of the timing giving the global maximum, and the condition at the boundary is $i(t_p^{\text{after}}) = i(t_p^{\text{during}})$, that is,

$$\Delta r = -\frac{\beta_{\text{off}}}{\beta_{\text{on}}} r(t_{\text{on}}) + \frac{\gamma}{\beta_{\text{on}}} \left[ 1 + \frac{\beta_{\text{off}} \log \left( \frac{\beta_{\text{on}}}{\gamma} \right) - \beta_{\text{on}} \log \left( \frac{\beta_{\text{off}}}{\gamma} \right)}{\beta_{\text{off}} - \beta_{\text{on}}} \right]. \quad \text{(S31)}$$

This condition gives the boundary between regions (i) and (ii) in Fig. 2(F) in the main text. For $\Delta r$ smaller than this condition, the peak of the second wave is larger than that during the intervention.

Boundaries between (i) and (iii)

Two peaks at the onset of the intervention and after the intervention can coexist. The maximum fraction of infected individuals is

$$i_{\max} = \max[i(t_{\text{on}}), i(t_p^{\text{after}})] \tag{S32}$$

$$= \max\left\{e^{-\frac{\beta_{\text{off}}}{\gamma} r(t_{\text{on}})} + r(t_{\text{on}}), \frac{\beta_{\text{off}} - \beta_{\text{on}}}{\beta_{\text{off}}}[r(t_{\text{off}}) - r(t_{\text{on}})] + \frac{\gamma}{\beta_{\text{off}}}\left[1 + \log\left(\frac{\beta_{\text{off}}}{\gamma}\right)\right]\right\}. \tag{S33}$$

The condition for the boundary $i(t_{\text{on}}) = i(t_p^{\text{after}})$ can be rewritten as

$$\Delta r = \frac{\beta_{\text{off}}}{\beta_{\text{off}} - \beta_{\text{on}}}\left[e^{-\frac{\beta_{\text{off}}}{\gamma} r(t_{\text{on}})} + r(t_{\text{on}})\right] - \frac{\gamma}{\beta_{\text{off}} - \beta_{\text{on}}}\left[1 + \log\left(\frac{\beta_{\text{off}}}{\gamma}\right)\right]. \tag{S34}$$

This boundary is shown in Figs. 2(F) and 3(F) in the main text.

Boundaries between (ii) and (iii)

Peaks at the onset and during the intervention cannot coexist. As shown in Eqs. (S21) and (S25),

$$r(t_{\text{on}}) = \frac{\gamma}{\beta_{\text{off}}} \log\left(\frac{\beta_{\text{on}}}{\gamma}\right), \tag{S35}$$

gives the boundary between regions (ii) and (iii). This condition does not depend on $\Delta r$ (Fig. 2(F) in the main text).

Boundaries between (iii) and (iv)

As shown in Eqs. (S25) and (S27),

$$r(t_{\text{on}}) = \frac{\gamma}{\beta_{\text{off}}} \log\left(\frac{\beta_{\text{off}}}{\gamma}\right), \tag{S36}$$

gives the condition for the boundary between regions (iii) and (iv). As in the case of the boundary between (ii) and (iii), this condition does not depend on $\Delta r$ (Figs. 2(F) and 3(F) in the main text).

## S5 Derivation of the final size equation with the intervention

As discussed in Eq. (S13), the relationship between $s(t_1)$ and $r(t_1)$ after the intervention at $t_1 > t_{\text{off}}$ is

$$s(t_1) = \exp\left[-\frac{\beta_{\text{off}}}{\gamma} r(t_1)\right] \exp\left\{\frac{\beta_{\text{off}} - \beta_{\text{on}}}{\gamma}\left[r(t_{\text{off}}) - r(t_{\text{on}})\right]\right\}. \tag{S37}$$

We obtain the final size equation for $t \to \infty$ with the intervention by substituting this into the condition $s(\infty) + r(\infty) = 1$ as

$$r(\infty) \;=\; 1 - \exp\left[-\frac{\beta_{\rm off}}{\gamma} r(\infty)\right] \exp\left\{\frac{\beta_{\rm off} - \beta_{\rm on}}{\gamma}\left[r(t_{\rm off}) - r(t_{\rm on})\right]\right\}, \quad \text{(S38)}$$

which is Eq. (6) in the main text.

## S6 Features of the final size equation

Let us study the features of Eq. (S38). Note that we assume that $R_{0,\rm on}$ is fixed in this section.

First, let us discuss the linear stability of the final state where $i(\infty) = 0$ and $r(\infty) = 1 - s(\infty)$ holds. The linearized equation around this state is given by

$$\frac{d\Delta s}{dt} \;=\; -\beta_{\rm off}[1 - r(\infty)]\Delta i, \quad \text{(S39)}$$

$$\frac{d\Delta i}{dt} \;=\; \{\beta_{\rm off}[1 - r(\infty)] - \gamma\}\Delta i, \quad \text{(S40)}$$

where $\Delta s = s(t) - [1 - r(\infty)]$, and $\Delta i$ denote the small perturbation to the final state. Note that the evolution equation for $\Delta r$ is eliminated from this equation, because the perturbation is restricted by the conservation $s(t) + i(t) + r(t) = 1$. Evidently, eigenvalues of the Jacobian matrix for the above linearized equation are zero and $\beta_{\rm off}[1 - r(\infty)] - \gamma$. The eigenvector component corresponding to the zero eigenvalue is $\Delta i = 0$, corresponding to the fact that any state $i = 0$ is an equilibrium and the perturbation to this state keeping $i = 0$ leads to another equilibrium. The sign of the other eigenvalue $\beta_{\rm off}[1 - r(\infty)] - \gamma$ determines the linear stability of the equilibrium. It turns out that the equilibrium is linearly stable if $s \geq 1/R_{0,\rm off}$ holds.

Next, we show that the final size monotonically decreases with respect to $r(t_{\rm off})$ for a fixed $r(t_{\rm on})$. Regareing Eq. (S38) as an implicit function of $r(\infty)$ and $\Delta r$, we obtain

$$\left\{1 - \frac{\beta_{\rm off}}{\gamma}[1 - r(\infty)]\right\}\frac{\partial r(\infty)}{\partial(\Delta r)} \;=\; -\frac{\beta_{\rm off} - \beta_{\rm on}}{\gamma}[1 - r(\infty)]. \quad \text{(S41)}$$

In the final state, the inequality $0 \leq 1 - r(\infty) \leq \gamma/\beta_{\rm off}$ must hold because of the herd immunity condition. Therefore,

$$\frac{\partial r(\infty)}{\partial(\Delta r)} \;\leq\; 0, \quad \text{(S42)}$$

holds. As $\Delta r$ monotonically increases with respect to $t_{\rm off}$ for a fixed $t_{\rm on}$, the final size is smaller if the intervention is longer. Equivalently, $r(\infty)$ monotonically increases with respect to $r(t_{\rm off})$ for a fixed $r(t_{\rm on})$.

Note that there exists the upper bound in $r(t_{\rm off})$. Let this upper bound be $\tilde{r}(r(t_{\rm on}))$. It satisfies Eq. (7) in the main text:

$$\tilde{r} \;=\; 1 - \exp\left[-\frac{\beta_{\rm off} - \beta_{\rm on}}{\gamma} r(t_{\rm on})\right] \exp\left[-\frac{\beta_{\rm on}}{\gamma}\tilde{r}\right], \quad \text{(S43)}$$

which is obtained for $\tilde{r} = r(\infty)$ in Eq. (S38). This upper bound is observed in Figs. 2(F) and 3(F) in the main text.

Finally, let us discuss $r(t_{\rm on})$ and $r(t_{\rm off})$ which minimizes the final size with fixed $R_{0,\rm on}$. The monotonicity of $r(\infty)$ with respect to $r(t_{\rm off})$ implies that the lower bound of the final size $r(\infty)$ with fixed $r(t_{\rm on})$ is obtained if $r(t_{\rm off})$ is replaced with $\tilde{r}(r(t_{\rm off}))$. Therefore, we focus on the final size equation in which $r(t_{\rm off})$ is replaced with $\tilde{r}$,

$$r(\infty) \quad = \quad 1 - \exp\left[-\frac{\beta_{\rm off}}{\gamma} r(\infty)\right] \exp\left\{\frac{\beta_{\rm off} - \beta_{\rm on}}{\gamma}\left[\tilde{r}(r(t_{\rm on})) - r(t_{\rm on})\right]\right\}. \tag{S44}$$

To minimize $r(\infty)$ in this equation, we should maximize $\tilde{r}(r(t_{\rm on})) - r(t_{\rm on})$ with respect to $r(t_{\rm on})$. This condition is given by

$$\frac{\partial}{\partial r(t_{\rm on})}[\tilde{r} - r(t_{\rm on})] \quad = \quad \frac{(\beta_{\rm off} - \beta_{\rm on})(1 - \tilde{r})}{\gamma - \beta_{\rm on}(1 - \tilde{r})} - 1 \tag{S45}$$

$$= \quad 0. \tag{S46}$$

This can be satisfied for

$$\tilde{r} \quad = \quad 1 - \frac{\gamma}{\beta_{\rm off}} = 1 - \frac{1}{R_{0,\rm off}}, \tag{S47}$$

$$r(t_{\rm on}) \quad = \quad \frac{\gamma}{\beta_{\rm off} - \beta_{\rm on}}\left[\log\left(\frac{\beta_{\rm off}}{\gamma}\right) - \beta_{\rm on}\left(\frac{\beta_{\rm off} - \gamma}{\gamma\beta_{\rm off}}\right)\right]. \tag{S48}$$

By substituting these equations into Eq. (S44), we obtain the final size equation:

$$r(\infty) \quad = \quad 1 - \frac{\gamma}{\beta_{\rm off}} \exp\left[\frac{\beta_{\rm off}}{\gamma} - 1\right] \exp\left[-\frac{\beta_{\rm off}}{\gamma} r(\infty)\right]. \tag{S49}$$

Interestingly, this transcendental equation has the solution $r(\infty) = \tilde{r} = 1 - \frac{1}{R_{0,\rm off}}$ if $R_{0,\rm on} < R_{0,\rm off} \log(R_{0,\rm off})/(R_{0,\rm off} - 1)$ (Eq. (8) in the main text). In other words, one can achieve herd immunity with the theoretically minimal removed fraction by intervening once if the intervention reproduction number is sufficiently small.

## S7 Numerical results for the final size

Figures 2(B)–(E) and 3(B)–(E) in the main text present the numerical results of the final size $r(\infty)$ by varying the intervention onset time $t_{\rm on}$ with the constant basic reproduction number without the intervention $R_{0,\rm off} = 2$ and intervention duration $\Delta t = 60$ days. As suggested theoretically in Section S6, the final size with the interventions (Figs. 2(B)–(E), 3(B)–(E) in the main text) is smaller than that without the intervention (Fig. 2(A) and 3(A) in the main text).

The final size is minimized in intermediate $t_{\rm on}$ in both cases (Figs. 2(D) and 3(D) in the main text). This result is intuitively understood as follows. The second wave occurs if the intervention onset is early (Figs. 2(B) and 3(B) in the main text). Conversely, the removed fraction is sufficiently enough before the intervention and the second wave is not observed if the onset is late (Figs. 2(E) and 3(E) in the main text).

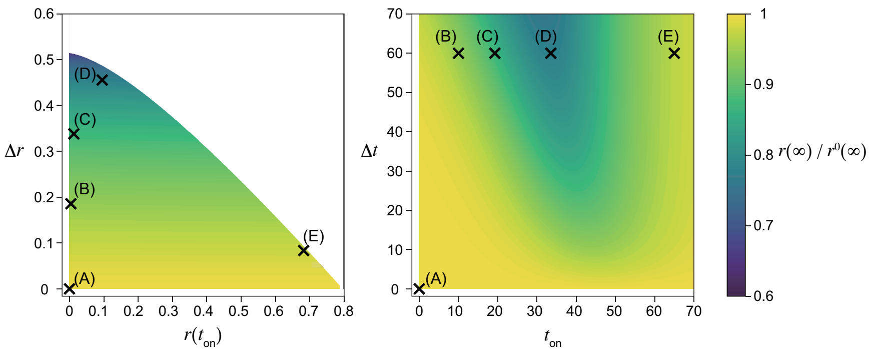


**Figure S 2** Final size with the intervention with large $R_{0,\mathrm{on}} = 1.4$. Parameters are $\beta_{\mathrm{off}} = 2/7$ days$^{-1}$, $\beta_{\mathrm{on}} = 1.4/7$ days$^{-1}$, $\gamma = 1/7$ days$^{-1}$, which are the same as those presented in Fig. 2 in the main text. The final size of the outbreak for each case is represented by $r(\infty)$, which is normalized by that without the intervention, $r^0(\infty)$. Left and right figures are plotted in terms of $r(t_{\mathrm{on}})$ and $\Delta r$, and $t_{\mathrm{on}}$ and $\Delta t$, respectively. Symbols (A)-(E) in the figures denote the intervention timings of the time series of those corresponding to Figs. 2(A)-(E) in the main text.

For sufficiently large $\Delta t$ and small enough $R_{0,\mathrm{on}}$, the final size reaches close to $1 - \frac{1}{R_{0,\mathrm{off}}} = 0.5$, which is the lower bound of the final size necessary to achieve herd immunity, for $r(t_{\mathrm{on}}) \approx 0.295$ (Fig. 3(D) in the main text), which corresponds to $t_{\mathrm{on}} = 42.681$ days. It should be noted that a maximum of $\Delta r = r(t_{\mathrm{off}}) - r(t_{\mathrm{on}})$ can be found for the intermediate $r(t_{\mathrm{on}})$ (Figs. S2 (left) and S3 (left)). Equation (6) in the main text (Eq. (S38)) suggests that a larger $\Delta r$ yields a smaller $r(\infty)$. Because $\tilde{r}$ is largest at intermediate $t_{\mathrm{on}}$, the final size is smallest at intermediate $t_{\mathrm{on}}$ (see Section S6).

As suggested by Eq. (S38), the final size depends on $\Delta r$ but is independent of $r(t_{\mathrm{on}})$ (Figs. S2 (left), S4(B), S3 (left), and S5(B)).

*Weak intervention (large $R_{0,\mathrm{on}} = 1.4$)*

The final size can be minimized with $t_{\mathrm{on}} = 0$, the early implementation of the intervention, in this case (Fig. S2). The timing of the intervention that minimizes the final size is different from that which minimizes the maximum fraction of infected individuals. In general, the optimal timing of the intervention depends on the objective function to be minimized. The theoretical prediction in Eq. (6) (Eq. (S38)), that the final size depends on $\Delta r$, is numerically verified (Fig. S2 (left)). For a constant intervention duration $\Delta t$ (Fig. S2 (right)), the final size is minimized with intermediate $t_{\mathrm{on}}$ (Fig. 2(D) in the main text).

*Strong intervention (small $R_{0,\mathrm{on}} = 0.7$)*

The simulation results (Fig. 3(B)–(E) in the main text) suggest that the final size is minimized with an intermediate starting time for small $R_{0,\mathrm{on}}$. Indeed, as shown in Fig. S3, the final size can be minimized at $t_{\mathrm{on}} \neq 0$. The minimized final size is closer to $r(\infty) = 1 - \frac{1}{R_{0,\mathrm{off}}} = 0.5$, which is the minimum fraction of removed individuals necessary to achieve herd immunity, compared with the case of $R_{0,\mathrm{on}} = 1.4$ (Fig. 3(D) in the main text).

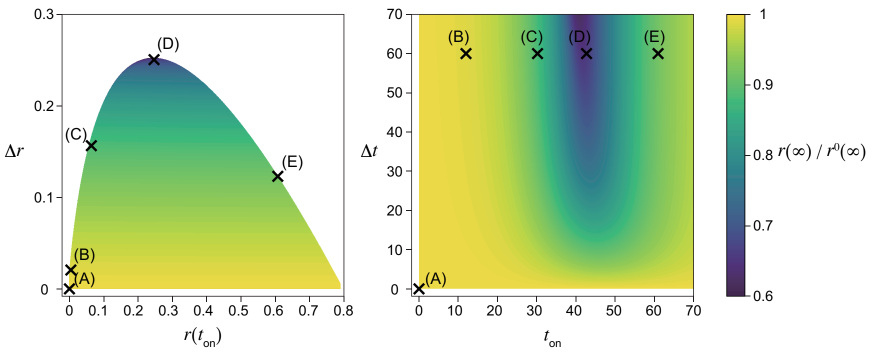


**Figure S 3** Final size with the intervention with a large $R_{0,\mathrm{on}} = 0.7$. Parameters are $\beta_{\mathrm{off}} = 2/7$ days$^{-1}$, $\beta_{\mathrm{on}} = 0.7/7$ days$^{-1}$, $\gamma = 1/7$ days$^{-1}$, which are the same as those in Fig. 3 in the main text. The final size of the outbreak for each case is represented by $r(\infty)$, which is normalized by that without the intervention, $r^0(\infty)$. Left and right figures are plotted using $r(t_{\mathrm{on}})$ and $\Delta r$, and $t_{\mathrm{on}}$ and $\Delta t$, respectively. Symbols (A)-(E) in the figures denote the intervention timings of the time series of those corresponding to Figs. 3(A)-(E) in the main text.

## S8 Contours of $i_{\max}$ and $r(\infty)$

As mentioned in the main text, the maximum fraction of infected individuals $i_{\max}$ depends linearly on $\Delta r$ if it appears after the intervention (case (i)). If the maximum appears during or at the onset of the intervention (cases (ii) and (iii)), $i_{\max}$ depends on $r(t_{\mathrm{on}})$ and does not depend on $\Delta r$. Therefore, the contours in the $(r(t_{\mathrm{on}}), \Delta r)$ plane are the horizontal and vertical lines for the former and latter cases, respectively. To visualize them explicitly, Figs. S4(A) and S5(A) plots the five contours for $i_{\max}$. As a theoretical prediction, they consist of horizontal and vertical lines.

Equation (S38) theoretically predicts that the final size $r(\infty)$ with the intervention depends on $\Delta r$ only, which implies that the contours are horizontal lines (Figs. S4(B) and S5(B)).

## S9 Dependence of $\bar{i}_{\max}$ on $R_{0,\mathrm{on}}$

We want to minimize the maximum fraction of infected individuals $\bar{i}_{\max}$ for a fixed $R_{0,\mathrm{on}}$ by varying $t_{\mathrm{on}}$ and $t_{\mathrm{off}}$. Note that $i_{\max}$ is decreasing function in $\Delta r$ (Eq. (S9)) for case (i), and $i_{\max}$ does not depend on $\Delta r$ in regions (ii) and (iii). Therefore, for a fixed $r(t_{\mathrm{on}})$, $\bar{i}_{\max}$ is achieved either in region (ii) or (iii). Here, $\bar{i}_{\max}$ can be analytically or semi-analytically calculated by evaluating $i_{\max}$ on the boundaries between (i) and (ii) or between (i) and (iii). We show below that the dependence of $\bar{i}_{\max}$ on $R_{0,\mathrm{on}}$ changes, in which the boundary gives $\bar{i}_{\max}$, leading to its non-trivial dependence on $R_{0,\mathrm{on}}$.

### Weak intervention ($R_{0,\mathrm{on}} \geq R^*_{0,\mathrm{on}} \approx 1.23$)

For large $R_{0,\mathrm{on}}$, $\bar{i}_{\max}$ is achieved at the boundary between regions (i) and (ii), where the peaks of the infected individuals during and after the intervention are equal (Fig. 2(F) in the main text). To discuss the parameter regions where this boundary exists, it is necessary to take into account the upper bound of removed individuals $\tilde{r}(r(t_{\mathrm{on}}))$. If $r(t_{\mathrm{off}}) = r(t_{\mathrm{on}}) + \Delta r$ at the boundary between regions (i) and (ii) given by Eq. (S31) is larger than $\tilde{r}$, this boundary is not observed at $t_{\mathrm{on}}$. In particular, it should be verified whether $\tilde{r}(0)$ is less than $\Delta r$ in Eq. (S31) for $r(t_{\mathrm{on}}) = 0$. Let $R^*_{0,\mathrm{on}}$

be the value of $R_{0,\mathrm{on}}$, below which the boundary between (i) and (ii) does not exist. For $R_{0,\mathrm{on}} \leq R^*_{0,\mathrm{on}}$, the boundary crosses $\tilde{r}$ at a non-zero intervention onset time $r(t_{\mathrm{on}})$, because the boundary is decreasing in $r(t_{\mathrm{on}})$ (Eq. (S31)) and $\tilde{r}$ is increasing in $r(t_{\mathrm{on}})$ for small $r(t_{\mathrm{on}})$.

For $R_{0,\mathrm{on}} \geq R^*_{0,\mathrm{on}}$, where there is a boundary between regions (i) and (ii) at $t_{\mathrm{on}} = 0$ (Fig. 2(F) in the main text), $\Delta r$ can be the largest at this boundary at $t_{\mathrm{on}} = 0$. Equation (S7) suggests that a larger $\Delta r$ yields a smaller $i_{\max}$, and $\bar{i}_{\max}$ is given by substituting $r(t_{\mathrm{on}}) = 0$ into Eq. (S20) as follows:

$$\bar{i}_{\max} = 1 - \frac{1}{R_{0,\mathrm{on}}}\Big[1 + \log(R_{0,\mathrm{on}})\Big]. \tag{S50}$$

This equation is used as the theoretical curve for $R_{0,\mathrm{on}} \geq R^*_{0,\mathrm{on}}$ in Fig. 4 in the main text. This equation implies that $\bar{i}_{\max}$ is increasing in $R_{0,\mathrm{on}}$ for $R_{0,\mathrm{on}} \geq R^*_{0,\mathrm{on}}$. See the next subsection for the value of $R^*_{0,\mathrm{on}}$.

### Medium-intervention ($R^*_{0,\mathrm{on}} \geq R_{0,\mathrm{on}} \geq R^{(1)}_{0,\mathrm{on}} \approx 1.08$)

If $R_{0,\mathrm{on}}$ is smaller than $R^*_{0,\mathrm{on}}$, region (ii) does not give the global maximum of the fraction of infected individuals for $t_{\mathrm{on}} = 0$. As a result, a larger $\Delta r$, which indicates a smaller $i_{\max}$, is realized for $t_{\mathrm{on}} \neq 0$. The upper bound of $r(t_{\mathrm{off}})$ at the boundary between regions (i) and (ii) equals $\tilde{r}(r(t_{\mathrm{on}}))$. Substituting the condition for the boundary between (i) and (ii) (Eq. (S31)) in the limit $r(t_{\mathrm{off}}) \to \tilde{r}$ into the condition for $\tilde{r}$ (Eq. (S43)), we obtain

$$\tilde{r} = 1 - e^{-A}, \tag{S51}$$

where

$$A := 1 + \frac{1}{R_{0,\mathrm{off}} - R_{0,\mathrm{on}}}\big[R_{0,\mathrm{off}}\log(R_{0,\mathrm{on}}) - R_{0,\mathrm{on}}\log(R_{0,\mathrm{off}})\big]. \tag{S52}$$

Substituting this into Eq. (S31) again, $r(t_{\mathrm{on}})$ at the boundary can be explicitly given as

$$r(t_{\mathrm{on}}) = \frac{R_{0,\mathrm{on}}}{R_{0,\mathrm{on}} - R_{0,\mathrm{off}}}\left[(1 - e^{-A}) - \frac{1}{R_{0,\mathrm{on}}}A\right]. \tag{S53}$$

Substituting these into Eq. (S7) or (S8), we obtain

$$\bar{i}_{\max} = e^{-A} - \frac{1}{R_{0,\mathrm{off}} - R_{0,\mathrm{on}}}\log\left(\frac{R_{0,\mathrm{off}}}{R_{0,\mathrm{on}}}\right), \tag{S54}$$

which is the theoretical curve for $R^*_{0,\mathrm{on}} \geq R_{0,\mathrm{on}} \geq R^{(1)}_{0,\mathrm{on}}$ in Fig. 4 in the main text. Note that $\bar{i}_{\max}$ is a decreasing function in $R_{0,\mathrm{on}}$. Therefore, $\bar{i}_{\max}$ is minimized at $R_{0,\mathrm{on}} = R^*_{0,\mathrm{on}}$. The condition for $R^*_{0,\mathrm{on}}$ is obtained by equating Eqs. (S50) and (S54). It is difficult to calculate $R^*_{0,\mathrm{on}}$ explicitly, and it was numerically obtained as $R^*_{0,\mathrm{on}} \approx 1.23$ in the present parameter setting.

Strong intervention ($R_{0,\mathrm{on}} \leq R_{0,\mathrm{on}}^{(1)}$)

For small $R_{0,\mathrm{on}}$, $\bar{i}_{\max}$ is determined differently. It is understood that a peak in region (ii) does not give $i_{\max}$ for $R_{0,\mathrm{on}} \leq R_{0,\mathrm{on}}^{(1)}$, where $R_{0,\mathrm{on}}^{(1)} \approx 1.08$.

In this case, the boundary between regions (i) and (iii) satisfying $r(t_{\mathrm{off}}) = \tilde{r}(r(t_{\mathrm{on}}))$ gives the condition for $\bar{i}_{\max}$ (Fig. 3(F) in the main text). This condition cannot be solved explicitly with respect to $\bar{i}_{\max}$. Therefore, we give the parametric equations for $\bar{i}_{\max}$ and $R_{0,\mathrm{on}}$ in terms of $r(t_{\mathrm{on}})$. The parametric equation for $\bar{i}_{\max}$ is

$$\bar{i}_{\max} = 1 - e^{-\frac{\beta_{\mathrm{off}}}{\gamma} r(t_{\mathrm{on}})} - r(t_{\mathrm{on}}). \tag{S55}$$

Next, we derive the parametric equation for $R_{0,\mathrm{on}}$. To this end, let us rewrite (S34) as

$$\Delta r = \frac{B(r(t_{\mathrm{on}}))}{R_{0,\mathrm{off}} - R_{0,\mathrm{on}}}, \tag{S56}$$

where

$$B(r(t_{\mathrm{on}})) = R_{0,\mathrm{off}}[e^{-\frac{\beta_{\mathrm{off}}}{\gamma} r(t_{\mathrm{on}})} + r(t_{\mathrm{on}})] - \Big[1 + \log(R_{0,\mathrm{off}})\Big]. \tag{S57}$$

Substituting this into Eq. (S43), we obtain a transcendental equation for $\tilde{r}$:

$$\tilde{r} = 1 - \exp[-R_{0,\mathrm{off}}\tilde{r}] \exp[B(r(t_{\mathrm{on}}))], \tag{S58}$$

which can be solved as

$$\tilde{r} = 1 + \frac{1}{R_{0,\mathrm{off}}} W_{-1}\Big( - R_{0,\mathrm{off}} \exp[B(r(t_{\mathrm{on}})) - R_{0,\mathrm{off}}]\Big), \tag{S59}$$

where $W_{-1}(\cdot)$ denotes the branch of the Lambert $W$ function which gives the real value other than the principal branch. Finally, we obtain the parametric equation for $R_{0,\mathrm{on}}$ as

$$R_{0,\mathrm{on}} = R_{0,\mathrm{off}} - \frac{B(r(t_{\mathrm{on}}))}{1 + \frac{1}{R_{0,\mathrm{off}}} W_{-1}(-R_{0,\mathrm{off}} \exp[B(r(t_{\mathrm{on}})) - R_{0,\mathrm{off}}]) - r(t_{\mathrm{on}})}. \tag{S60}$$

Equations (S55) and (S60) are used to plot the theoretical curves for $R_{0,\mathrm{on}} \leq R_{0,\mathrm{on}}^{(1)}$ in Fig. 4 in the main text.

## S10 Optimization problems with respect to final size

Two scenarios for the optimization problems in the hybrid nonlinear dynamical systems are presented in the main text. Here, we discuss the problems using the final size as the objective functions.

### Minimizing final size with a constraint in intervention duration

Targeting the final size to be minimized, we discuss two constraints based on Figs. S2 and S3. As in the case for the minimization problem of the maximum fraction of infected individuals, the final size is minimized at an intermediate $t_{\mathrm{on}}$ with the constraint of a constant $\Delta t$ (Figs. 2(D) and 3(D) in the main text, and Figs. S2 (right) and S3 (right)). Note that this $t_{\mathrm{on}}$ is different from the one which minimizes $i_{\mathrm{max}}$. This result is intuitively understood in the right panels of these figures. The contour of the final size depends on $\Delta r$. For a constant intervention duration, $\Delta r$ is largest with intermediate $r(t_{\mathrm{on}})$, which maximizes the final size with this constraint.

### Minimizing intervention duration with a constraint on the final size

It is possible to consider this rule to minimize the intervention duration along the contour of the final size in Figs. S2 and S3. This corresponds to intervening while keeping $\Delta r$ constant. There exists an intermediate $t_{\mathrm{on}}$ that minimizes the final size in this setting as well.

## S11 Effect of continuous change of $\beta$ at the onset and offset of the NPI

As discussed in the main text, it takes a finite time for the response of people to NPIs, which has been observed in a real situation [6]. Here, we see that the finite time interval of the change in the parameter $\beta$ from $\beta_{\mathrm{off}}$ to $\beta_{\mathrm{on}}$ gives a correction to the results presented in this paper. Let $\tau$ be the time interval that $\beta$ changes from $\beta_{\mathrm{off}}$ to $\beta_{\mathrm{on}}$ when the NPI is implemented. Here, let us discuss the case where $\beta$ changes linearly in time between $t_{\mathrm{on}}$ and $t_{\mathrm{on}}+\tau$, namely,

$$\beta(t) \quad = \quad \beta_{\mathrm{off}} + \frac{\beta_{\mathrm{on}}-\beta_{\mathrm{off}}}{\tau}(t-t_{\mathrm{on}}), \tag{S61}$$

where $t_{\mathrm{on}} \le t \le t_{\mathrm{on}}+\tau$ (Fig. S6).

Let us discuss the effect of the finite $\tau$ compared with the case for $\tau = 0$, i.e., discontinuous change from $\beta_{\mathrm{off}}$ to $\beta_{\mathrm{on}}$ studied in the other parts of this paper. If $\beta$ switches instantaneously from $\beta_{\mathrm{off}}$ to $\beta_{\mathrm{on}}$, $s(t_{\mathrm{on}}+\tau)$ is given by

$$s(t_{\mathrm{on}}+\tau) \quad = \quad \exp\left[-\frac{\beta_{\mathrm{on}}}{\gamma}r(t_{\mathrm{on}}+\tau)\right]\exp\left[-\frac{\beta_{\mathrm{off}}-\beta_{\mathrm{on}}}{\gamma}r(t_{\mathrm{on}})\right]. \tag{S62}$$

Our interest here is to see how this equation is modified if $\beta$ changes in the finite interval.

Let us denote $s'(t)$, $i'(t)$, and $r'(t)$ for the fraction of each state for finite $\tau$ to distinguish from the results for $\tau = 0$. Then, Eqs. (S1)-(S3) are rewritten as

$$\frac{ds'}{dt} \quad = \quad -\left[\beta_{\mathrm{off}} + \frac{\beta_{\mathrm{on}}-\beta_{\mathrm{off}}}{\tau}(t-t_{\mathrm{on}})\right]s'i', \tag{S63}$$

$$\frac{di'}{dt} \quad = \quad \left[\beta_{\mathrm{off}} + \frac{\beta_{\mathrm{on}}-\beta_{\mathrm{off}}}{\tau}(t-t_{\mathrm{on}})\right]s'i' - \gamma i', \tag{S64}$$

$$\frac{dr'}{dt} \quad = \quad \gamma i', \tag{S65}$$

where $t_{\rm on} \le t \le t_{\rm on}+\tau$. Equation (S63) is integrated with $s'(t_{\rm on}) = s(t_{\rm on}) = \exp[-\beta_{\rm off} r(t_{\rm on})/\gamma]$, $i'(t_{\rm on}) = i(t_{\rm on})$, and $r'(t_{\rm on}) = r(t_{\rm on})$, leading to

$$\begin{aligned} s'(t_{\rm on}+\tau) &= s(t_{\rm on}) \exp\left\{ -\int_{t_{\rm on}}^{t_{\rm on}+\tau} \left[\beta_{\rm off} + \frac{\beta_{\rm on}-\beta_{\rm off}}{\tau}(t'-t_{\rm on})\right] i'(t')dt' \right\} && \text{(S66)} \\ &= s(t_{\rm on}) \exp\left\{ -\int_{t_{\rm on}}^{t_{\rm on}+\tau} \left[\beta_{\rm off} + \frac{\beta_{\rm on}-\beta_{\rm off}}{\tau}(t'-t_{\rm on})\right] \left(\frac{1}{\gamma}\frac{dr'}{dt'}\right) dt' \right\} && \text{(S67)} \\ &= s(t_{\rm on}) \exp\left\{ -\frac{\beta_{\rm off}}{\gamma}[r'(t_{\rm on}+\tau) - r(t_{\rm on})] \right\} \exp\left[ -\frac{\beta_{\rm on}-\beta_{\rm off}}{\gamma} r'(t_{\rm on}+\tau)\right] \\ &\quad \times \exp\left\{ -\frac{\beta_{\rm off}-\beta_{\rm on}}{\gamma\tau} \int_{t_{\rm on}}^{t_{\rm on}+\tau} r'(t')dt' \right\} && \text{(S68)} \\ &= \exp\left[ -\frac{\beta_{\rm on}}{\gamma} r'(t_{\rm on}+\tau)\right] \exp\left\{ -\frac{\beta_{\rm off}-\beta_{\rm on}}{\gamma\tau} \int_{t_{\rm on}}^{t_{\rm on}+\tau} r'(t')dt' \right\}, && \text{(S69)} \end{aligned}$$

where the integration by parts is applied. Let us introduce the Taylor series of $r'(t)$,

$$r'(t) = r(t_{\rm on}) + \gamma i(t_{\rm on})(t-t_{\rm on}) + O((t-t_{\rm on})^2), \quad \text{(S70)}$$

where the coefficient in $O((t-t_{\rm on}))$ term is determined from Eq. (S65). Substituting this expansion into Eq. (S69), we obtain

$$\begin{aligned} s'(t_{\rm on}+\tau) &= \exp\left[ -\frac{\beta_{\rm on}}{\gamma} r'(t_{\rm on}+\tau)\right] \exp\left[ -\frac{\beta_{\rm off}-\beta_{\rm on}}{\gamma} r(t_{\rm on})\right] \\ &\quad \times \exp\left[ \tau \frac{\beta_{\rm off}-\beta_{\rm on}}{2} i(t_{\rm on}) + O(\tau^2)\right]. && \text{(S71)} \end{aligned}$$

Equation (S70) implies $r'(t_{\rm on}+\tau) - r(t_{\rm on}+\tau) = O(\tau^2)$, because the $O(\tau)$ term in the Taylor series does not depend on $\beta(t)$. Therefore, we have

$$s'(t_{\rm on}+\tau) = \exp\left[ -\frac{\beta_{\rm on}}{\gamma} r(t_{\rm on}+\tau)\right] \exp\left[ -\frac{\beta_{\rm off}-\beta_{\rm on}}{\gamma} r(t_{\rm on})\right] \left[1 + \tau \frac{\beta_{\rm off}-\beta_{\rm on}}{2} i(t_{\rm on})\right] + O(\tau^2), \quad \text{(S72)}$$

which gives the lowest order correction. This correction term vanishes for $\tau \to 0$, which recovers the results in this paper.

**Author details**

**References**


1. Kermack, W.O., McKendrick, A.G.: A contribution to the mathematical theory of epidemics. Proceedings of the royal society of london. Series A, Containing papers of a mathematical and physical character **115**(772), 700–721 (1927)
2. Anderson, R.M., May, R.M.: Infectious Diseases of Humans: Dynamics and Control. Oxford university press, Oxford (1992)
3. Chowell, G., Brauer, F.: The basic reproduction number of infectious diseases: computation and estimation using compartmental epidemic models. In: Mathematical and Statistical Estimation Approaches in Epidemiology, pp. 1–30. Springer, Dordrecht (2009)
4. Inaba, H.: Age-structured Population Dynamics in Demography and Epidemiology. Springer, Singapore (2017)
5. Kiss, I.Z., Miller, J.C., Simon, P.L.: Mathematics of Epidemics on Networks. Springer, Cham (2017)
6. Liu, Q.-H., Bento, A.I., Yang, K., Zhang, H., Yang, X., Merler, S., Vespignani, A., Lv, J., Yu, H., Zhang, W., *et al.*: The covid-19 outbreak in sichuan, china: epidemiology and impact of interventions. PLoS computational biology **16**(12), 1008467 (2020)

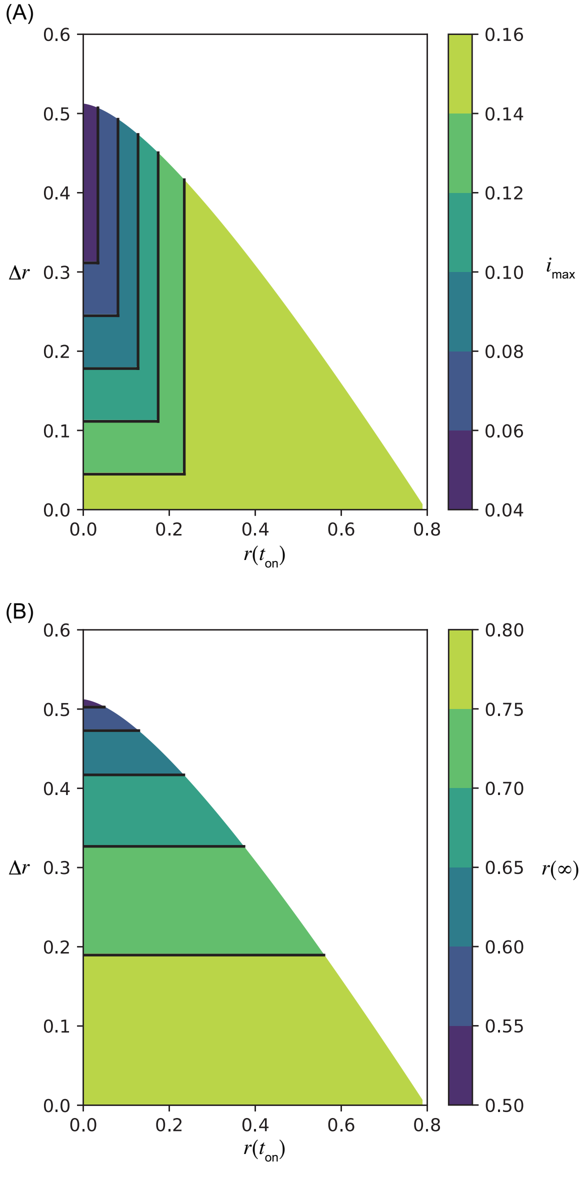


**Figure S 4** Contours of: (A) the maximum fraction of infected individuals, $i_{\max}$, and (B) the final size $r(\infty)$ for $R_{0,\text{on}} = 1.4$. The solid lines are obtained analytically. These figures are the same as Fig. 2(F) in the main text and Fig. S2(left) but with different visualizations.

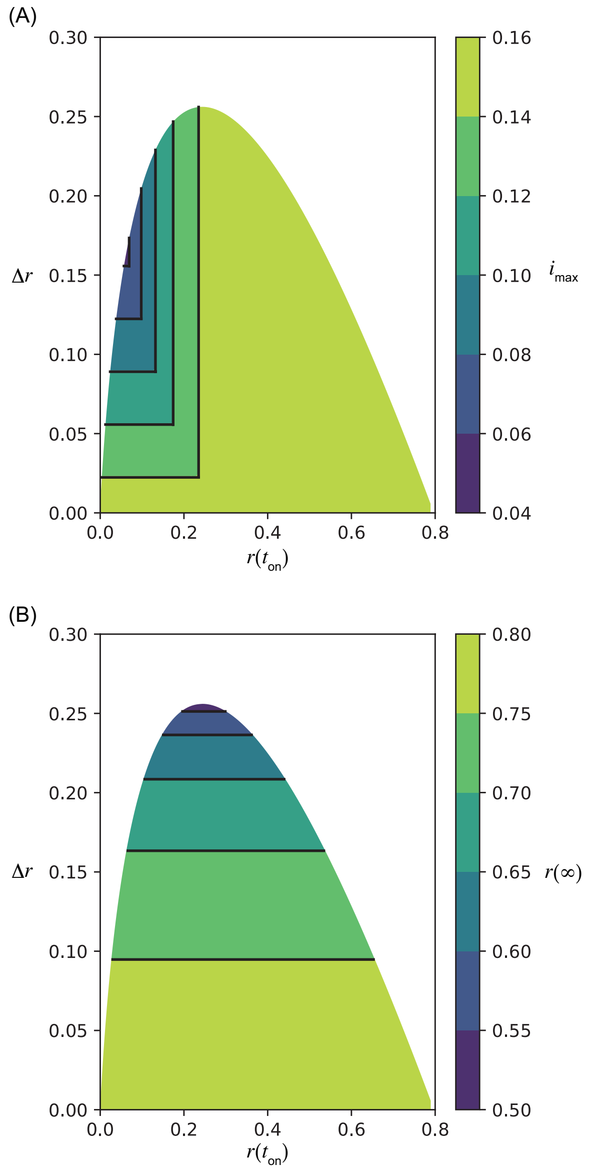


**Figure S 5** Contours of: (A) the maximum fraction of the infected individuals, $i_{\max}$, and (B) the final size, $r(\infty)$ for $R_{0,\mathrm{on}} = 0.7$. The solid lines are obtained analytically. These figures are the same as Figs. 3(F) in the main text and Fig. S3(left) but with different visualization.

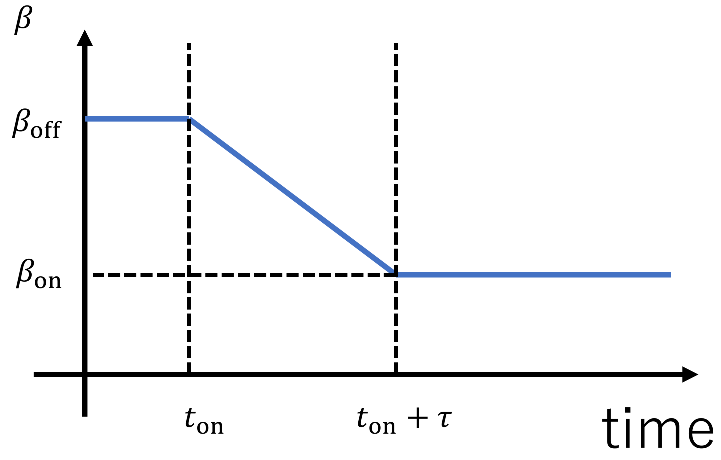


**Figure S 6** Case of continuous change of $\beta$ considered in Eq. (S61). Value of $\beta$ changes from $\beta_{\mathrm{off}}$ to $\beta_{\mathrm{on}}$ linearly in time.
